# Fixational Eye Movements Enhance the Precision of Visual Information Transmitted by the Primate Retina

**DOI:** 10.1101/2023.08.12.552902

**Published:** 2023-08-14

**Authors:** Eric G. Wu, Nora Brackbill, Colleen Rhoades, Alexandra Kling, Alex R. Gogliettino, Nishal P. Shah, Alexander Sher, Alan M. Litke, Eero P. Simoncelli, E.J. Chichilnisky

**Affiliations:** 1. Department of Electrical Engineering, Stanford University; 2. Department of Physics, Stanford University; 3. Department of Bioengineering, Stanford University; 4. Department of Neurosurgery, Stanford University; 5. Department of Ophthalmology, Stanford University; 6. Hansen Experimental Physics Laboratory, Stanford University; 7. Neurosciences PhD Program, Stanford University; 8. Santa Cruz Institute for Particle Physics, University of California, Santa Cruz; 9. Flatiron Institute, Simons Foundation; 10. Center for Neural Science, New York University; 11. Courant Institute of Mathematical Sciences, New York University

## Abstract

The retina transmits visual signals to the brain in the spiking activity of retinal ganglion cells (RGCs). This signal is necessarily imperfect: some visual information is lost in phototransduction and retinal processing. To quantify the transmitted visual signal, we developed a Bayesian method to reconstruct images from the simultaneously recorded spikes of hundreds of macaque RGCs of the four dominant types. The algorithm combines a stochastic likelihood model for RGC light responses that is fitted to spiking data, with a prior model for natural images implicitly embedded within an artificial neural network trained for image denoising. When applied to retinal population responses to both flashed images and images jittered to emulate fixational eye movements, the method provided reconstruction performance exceeding or matching all previous reconstruction algorithms, in an interpretable analytical framework that provided insight into the neural code. Reconstructions improved with increasing jitter amplitude over a behaviorally relevant range (even when the jitter trajectory was unknown), revealing that fixational eye movements improve rather than degrade the retinal signal. Reconstructions were degraded by artificial perturbation of spike times as small as 5 ms, revealing a temporal encoding precision finer than expected from previous studies. Ablating cell-to-cell interactions in the encoding model substantially reduced reconstruction quality, indicating the importance of stimulus-evoked correlations in representing the visual scene. Thus, fixational eye movements contribute to highly precise retinal population activity, enabling more accurate transmission of visual signals to the brain.

## Introduction

Vision begins with the retina, which transforms incoming light into electrical signals, processes these signals, and transmits them to the brain in the spiking activity of retinal ganglion cells (RGCs). This encoding process has been studied for nearly a century, with contemporary models capturing the details of RGC responses with a high degree of precision. But quantifying coding precision does not directly reveal how effectively the visual scene is conveyed by RGCs to the brain, nor how that effectiveness depends on spike timing and cell-to-cell correlations. Nor does it elucidate the degree to which the RGC code is specialized for the stimulus conditions that the visual system evolved to analyze: naturally-occurring patterns of light, with global image shifts arising from eye movements.

To probe the retinal code under these conditions, we develop and apply a novel method for reconstructing natural images and movies from the spiking activity of complete populations of RGCs recorded in the primate retina. Rather than fitting a model to directly map recorded RGC spikes to images [Warland 1997; Kim 2020, Brackbill 2020], we use a Bayesian formalism – combining a *likelihood* obtained from the retinal spikes with separately-acquired *prior* information about the statistical structure of natural images. Specifically, images are reconstructed by numerical optimization of the posterior density, arising from the product of (1) an image likelihood obtained from an encoding model fitted to RGC data [Pillow 2008] that captures the stochastic responses of RGCs to visual stimuli, and (2) a natural image prior implicit in an artificial neural network pre-trained on a natural image database to perform Gaussian denoising [Zhang 2021]. This approach confers unique advantages for analysis and interpretation of the retinal signals. We demonstrate that the method achieves state-of-the-art reconstruction performance, and then use it to demonstrate for the first time the importance of fixational eye movements, spike timing precision, and cell-to-cell correlations in the retinal code for natural visual stimuli.

## Results

To characterize the visual signals evoked by natural images, we recorded light responses of RGCs in isolated macaque retina with a large-scale multi-electrode array [Litke 2004]. This method captured the activity of nearly complete populations of several hundred RGCs of the four numerically dominant types (ON midget, OFF midget, ON parasol, OFF parasol), which comprise roughly 70% of RGC axons projecting to the brain [Field 2007]. Spatiotemporal white noise stimuli were used to identify cells and map their receptive fields [Field 2007, Rhoades 2019].

### Bayesian reconstruction of flashed images

**Figure 1.**
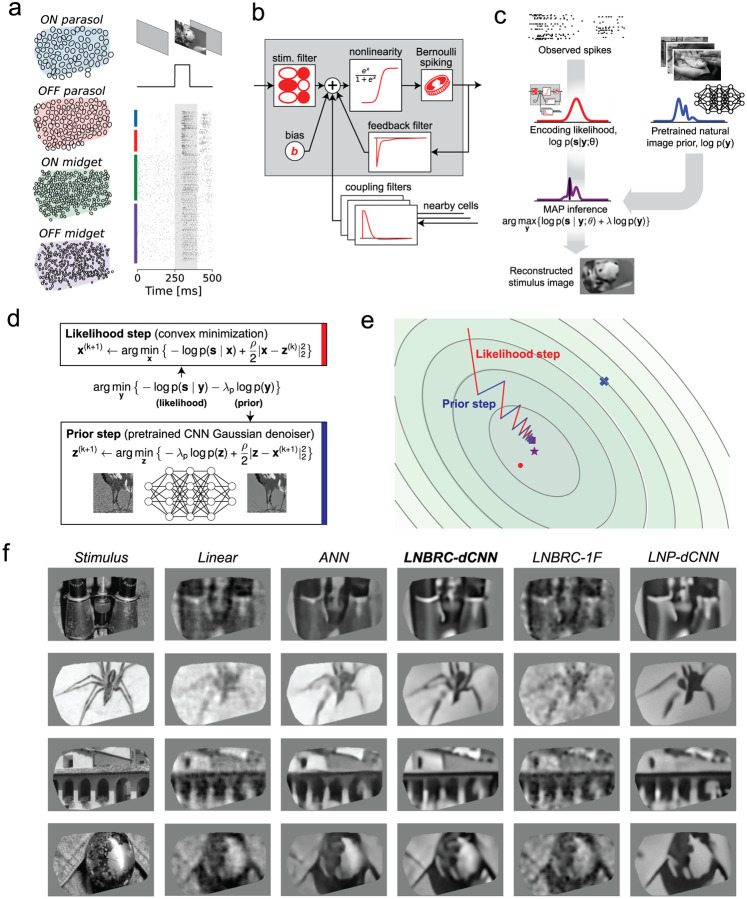
Reconstruction of flashed natural images from RGC spikes. (a) Example macaque retinal data. Receptive field mosaics for the major RGC types (ON parasol, OFF parasol, ON midget, OFF midget). Natural images are flashed for 100ms, and spikes recorded from all 691 cells over a 150 ms interval (gray region) were used for LNBRC model fitting and reconstruction. **(b)** LNBRC encoding model. Model cell responses are computed from the spatio-temporally filtered visual stimulus, combined with filtered spike trains from the cell and neighboring cells. These filtered spiking inputs capture both spike train temporal structure and cell-to-cell correlations. **(c)** Bayesian reconstruction. The likelihood computed using the LNBRC encoding model is combined with a separately trained natural image prior to produce a posterior density for the stimuli given observed spike trains. **(d)** Half-quadratic variable splitting algorithm for approximate MAP optimization. The method alternates between optimizing the likelihood (a convex minimization problem, solved using gradient descent), and optimizing the prior probability (by applying an artificial neural network pre-trained to perform Gaussian denoising on natural images). **(e)** Visualization of the optimization path for a highly-simplified two-dimensional toy problem (red lines are likelihood steps, blue lines are prior steps). The contours indicate level sets of the posterior, with mode of posterior (purple star), likelihood (red dot) and prior (blue x). The step size progressively decreases, corresponding to increasing values of schedule hyperparameter ρ. **(f)** Example reconstructions comparing LNBRC-dCNN with stimulus, benchmarks, and alternative models. Columns: *Stimulus*, the image presented to the retina; *Linear reconstruction*, a simple benchmark; *ANN*, direct artificial neural network reconstruction [Kim 2020]; *LNBRC-dCNN*, our Bayesian method; *LNBRC-1F*, Bayesian method with the dCNN image prior with a simpler 1/F Gaussian image prior; and *LNP-dCNN*, replacing the LNBRC likelihood with a simpler LNP likelihood.

We first examined reconstruction of images presented in brief flashes to the retina. Although the dynamics of the flashed stimulus differ markedly from natural vision, the simplicity of the stimulus enabled evaluation of the image reconstruction approach and comparison to previous methods. Thousands of grayscale photographic images from the ImageNet database [Fei-Fei 2009] were presented, for a duration of 100 ms with consecutive trials separated by 400 ms of uniform gray screen (Fig. 1a, also see Methods).

Flashed natural images were reconstructed from evoked RGC activity using an Bayesian approximate maximum *a posteriori* (MAP) algorithm (see [Wu 2022]). The posterior density (probability of an image given observed spikes) is the product of two separately defined and estimated components: (1) a *likelihood* model of the natural image stimulus *y* evoking the measured spiking response *s*, *p*(*s* | *y*), computed using a probabilistic encoding model of RGC spiking in response to natural image stimuli; (2) a *prior* model of natural images, *p*(*y*), obtained implicitly from a Gaussian-denoising neural network (Fig. 1c). The likelihood was computed from an encoding model that summed the effects of the visual input, spike history, and spike trains of nearby neurons (to capture spike train temporal structure and cell-to-cell correlations) and then transformed the output with an instantaneous sigmoidal nonlinearity to provide a firing probability for a Bernoulli spike generator (Fig. 1b). This model generalizes the commonly-used linear-nonlinear-Poisson (LNP) cascade model, replacing Poisson spiking with Bernoulli spiking (equivalent at fine time scales) and incorporating recursive feedback and coupling filters [Pillow 2008] – we refer to it as the Linear-Nonlinear-Bernoulli with Recursive Coupling (LNBRC) model. Model parameters (stimulus, feedback, and coupling filters, and an additive constant) were jointly fitted to recorded RGC data by maximizing the likelihood of observed spikes given the stimulus, augmented with regularization terms to induce sparsity in the filter weights (see Methods). Separately, an implicit image prior was obtained by training a denoising convolutional neural network (dCNN) to remove additive Gaussian noise from a large collection of natural images [Zhang 2021]. Such priors underlie the “diffusion models” [Sohl-Dickstein 2015] that represent the current state-of-the-art in machine learning for image synthesis [Song 2019, Ho 2020] and inference [Kadkhodaie 2020, Cohen 2021, Kawar 2021].

With these two components, the reconstruction procedure maximized the posterior by alternating between an encoding likelihood optimization step (solved with unconstrained convex minimization) and a prior optimization step (solved with a single forward pass of the denoiser [Venkatakrishnan 2013, Zhang 2021]) (Fig. 1d,e, see Methods), yielding an estimate of the most probable image given the RGC spikes and natural image statistics.

The performance of the MAP reconstruction algorithm was characterized qualitatively with visual image comparison and quantitatively with MS-SSIM [Wang 2004], a commonly used measure of perceptual image quality. Example reconstructions are shown in Fig. 1f. Reconstruction performance was qualitatively and quantitatively more accurate than that obtained using linear reconstruction [Rieke 1997, Warland 1997, Brackbill 2020] (mean MS-SSIM of 0.685, 0.652, 0.660, and 0.652 for LNBRC-dCNN MAP reconstructions in the four preparations tested, compared to 0.624, 0.616, 0.578, and 0.575 for linear reconstruction). Performance was comparable to state-of-the-art neural networks trained to nonlinearly recover the high spatial frequency components of images [Kim 2020] (mean MS-SSIM of 0.689, 0.683, 0.651, and 0.653, respectively). In addition to reconstruction quality, the MAP approach provided greater interpretability by separating the likelihood and prior components of estimation, and broader usability with limited retinal data (the retinal encoding models contained ~1.5 million parameters, in comparison with ~240 million parameters for the benchmark direct neural network method).

To examine the importance of the encoding and prior models, MAP reconstruction performance with the full model (labeled LNBRC-dCNN) was compared to that achieved with a simpler spectral Gaussian image prior (LNBRC-1F) or with a likelihood corresponding to a simpler LNP encoding model (LNP-dCNN). Images reconstructed using the full approach had sharper and more detailed image structure (edges, contours, textures) than those reconstructed using the 1/F prior, and contained more fine spatial detail than those reconstructed using the LNP encoding model (Fig. 1f). Quantitatively, reconstructions produced using LNBRC-dCNN exhibited greater similarity to the original image than those produced with the simpler 1/F prior or the simpler LNP encoding model (mean MS-SSIM of 0.685, 0.652, 0.660, and 0.652 across preparations using LNBRC-dCNN, in comparison with 0.612, 0.573, 0.577, and 0.565 using LNBRC-1/F, and 0.635, 0.613, 0.597, and 0.603 using LNP-dCNN). Thus, both the dCNN image prior and the LNBRC encoding model contribute substantially to producing high-quality natural image reconstructions.

### Bayesian reconstruction of images displayed with fixational eye movements

Fixational jitter (drift), the small but incessant eye movements that occur when fixating a visual target, is a fundamental component of natural vision in primates. These eye movements have been hypothesized to enhance visual resolution by sampling the image at many spatial phases relative to the lattice of RGC receptive fields [Patrick 2017, Ratnam 2017, Anderson 2020], and/or by modulating high frequency spatial details into the temporal domain [Rucci 2007, Kuang 2012]. However, psychophysical studies [Murakami 1998, Poletti 2010, Ratnam 2017] suggest that the visual system may not have precise knowledge of the eye position, opening the possibility that positional uncertainty could instead degrade the retinal signal [Packer 1992] (but see [Rucci 2015]). Although simulation studies [Pitkow 2007, Burak 2010, Ahissar 2012, Anderson 2020] have explored the possibility of using the retinal signal alone to compensate for fixational eye movements, it remains uncertain whether unknown eye jitter enhances or degrades the retinal representation. We directly characterized the effects of jitter eye movements by reconstructing images from the experimentally-recorded responses of RGCs to jittered natural stimuli.

**Figure 2.**
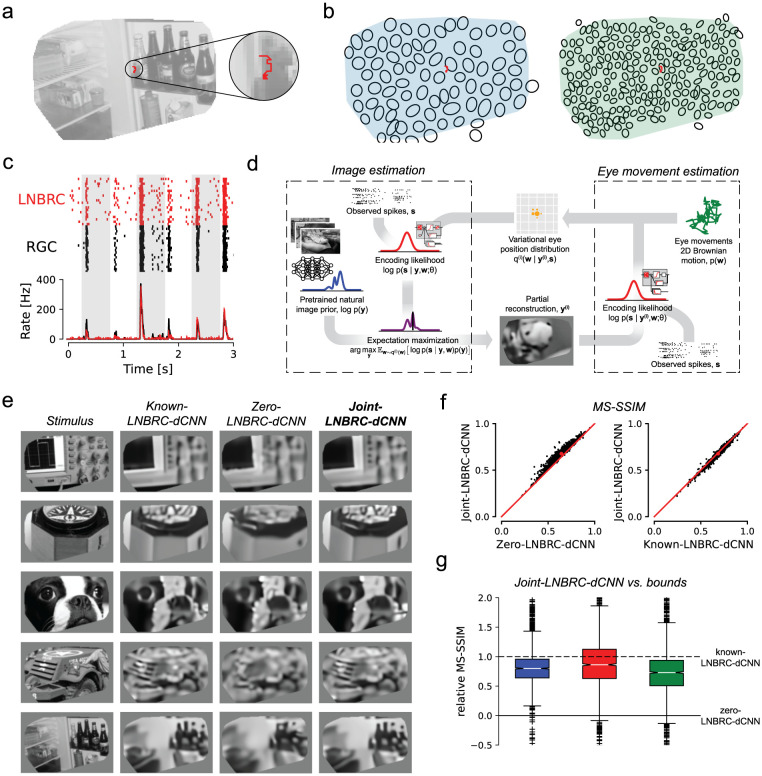
Reconstruction of jittered natural images from RGC spikes. **(a)** Example stimulus image (masked to include only the region covered by recorded cells), with an example jitter eye movement trajectory overlaid (red). **(b)** Example ON parasol receptive field mosaic (left) and ON midget mosaic (right), with example jitter trajectory (red). The simulated eye movements were typically comparable to the size of a midget RGC receptive field. **(c)** Top: comparison of spikes recorded from an example ON parasol RGC to repeated presentations of the same stimulus (black ticks) with simulated responses of the fitted LNBRC model (red ticks). Bottom: average spike rates over time corresponding to the above rasters. **(d)** Schematic of the joint-LNBRC-dCNN reconstruction algorithm: the algorithm alternates between an image estimation update step (left), in which the stimulus is reconstructed by using the LNBRC model and denoiser CNN image prior to maximize the expected log-posterior over a variational distribution for eye movements, and an eye movements update step (right), in which the variational distribution for eye movements is updated given the reconstructed image. **(e)** Example reconstructions for the eye movements stimulus, using LNBRC encoding model and dCNN prior. Columns: *Stimulus*, the image presented to the retina; *Known-LNBRC-dCNN*, MAP reconstruction with known eye movements; *Zero-LNBRC-dCNN*, MAP reconstruction with the (incorrect) assumption of zero eye movements; and *Joint-LNBRC-dCNN*, joint estimation of image and eye movements. **(f)** Left: Performance comparison between joint-LNBRC-dCNN and zero-LNBRC-dCNN. Reconstruction quality using joint-LNBRC-dCNN exceeded was better than that of zero-LNBRC-dCNN for nearly every image. Right: performance of joint-LNBRC-dCNN and known-LNBRC-dCNN. Reconstruction quality lies near the line of equality, with known-LNBRC-dCNN slightly outperforming joint-LNBRC-dCNN. **(g)** Performance of joint estimation procedure joint-LNBRC-dCNN, normalized relative to zero-LNBRC-dCNN (y=0, solid line) and known-LNBRC-dCNN (y=1, dashed line). The box in the plot marks the median and the inter-quartile range (IQR), while the whiskers extend to 1.5 times the IQR. Outliers are marked with a +. For all three preparations, the relative reconstruction quality for joint-LNBRC-dCNN was typically near 1 (mean values: 0.976, 1.02, and 0.793), the performance with known eye movements.

We measured RGC responses to movies consisting of images from the ImageNet database [Fei-Fei 2009], presented with randomly jittered spatial offsets in each frame to emulate fixational eye movements. Images were displayed for 500 ms, with each 8.33 ms frame spatially shifted relative to the previous frame according to a discretized sample from a 2D Gaussian distribution with a standard deviation of 10 μm (Fig 2ab), approximately matching the diffusion constant for fixational jitter eye movements in humans [Kuang 2012, Rucci 2015] and macaques [Z.M. Hafed and R.J. Krauzlis, personal communication, June 2008]. The LNBRC model was fitted to RGC responses to jittered stimuli by maximizing likelihood. Model fit quality was assessed by comparing the model-simulated spikes with recorded data (Fig. 2c), and by computing the fraction of response variance explained by the model. Although some small systematic deviations from the data were observed (Fig. 2c), in general the LNBRC model effectively captured responses to natural stimuli with fixational eye movements (Extended Data Fig 1).

The fitted LNBRC was combined with the dCNN natural image prior for simultaneous estimation of the stimulus image and eye position using a modified approximate MAP procedure. To avoid marginalization over the eye movement trajectories, an expectation-maximization (EM) algorithm [Anderson 2020] was used to alternate between reconstructing the intermediate image that maximized the expected log posterior over an estimated distribution of eye movement trajectories, and using that intermediate image to update the eye movement distribution (Fig. 2d, also Methods and Supplement).

The effectiveness of this procedure (labeled joint-LNBRC-dCNN) at compensating for unknown eye movements was evaluated by comparing reconstruction quality to the case in which eye movements were known exactly (known-LNBRC-dCNN), and the case in which eye movements were incorrectly assumed to be zero (zero-LNBRC-dCNN). Reconstruction quality for joint-LNBRC-dCNN exceeded that of zero-LNBRC-dCNN (mean MS-SSIM of 0.677, 0.652, and 0.638 for each preparation for joint-LNBRC-dCNN, in comparison with 0.642, 0.617, and 0.615 for zero-LNBRC-dCNN) and approached that of known-LNBRC-dCNN (mean MS-SSIM of 0.685, 0.656, and 0.646 for the same respective preparations). Notably, this held true for nearly every image evaluated, for every preparation (Fig. 2f–g). Qualitative comparisons (Fig. 2e) revealed that the joint solution recovered substantially more image structure and fine spatial detail than the one that ignored eye movements, and produced reconstructions that were similar in content and quality to those produced with known eye movements. These results demonstrate that compensation for jitter eye movements is critical for recovering fine spatial detail in the visual scene, and that the RGC spikes alone are sufficient to perform this compensation.

### Fixational eye movements enhance the retinal visual signal

**Figure 3.**
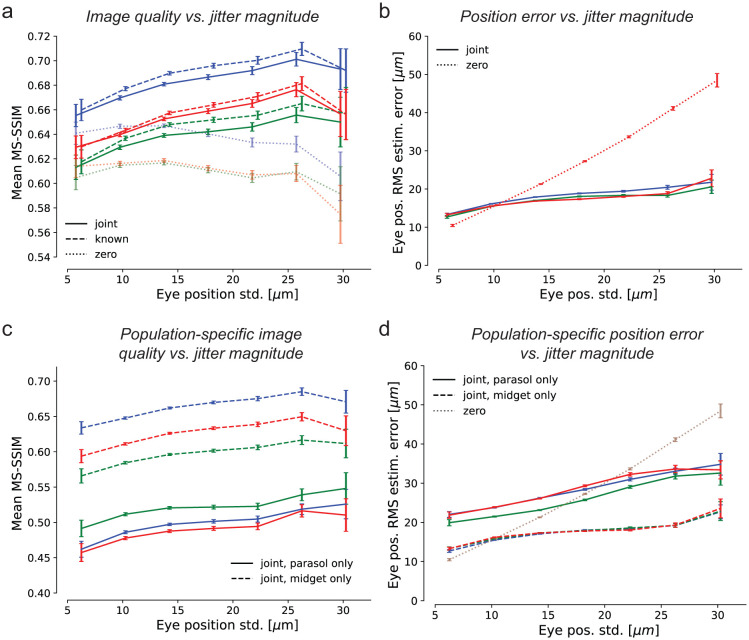
Effects of jitter magnitude on reconstruction quality. **(a)** Image reconstruction performance for three preparations (colors) as a function of the magnitude of eye movements simulated during the stimulus presentation, for joint-LNBRC-dCNN (solid line), known-LNBRC-dCNN (dashed line), and zero-LNBRC-dCNN (dotted line). In all preparations, reconstruction quality for joint-LNBRC-dCNN as well as known-LNBRC-dCNN increased with eye position jitter, up to (but not including) the largest eye movements tested. Reconstructions for zero-LNBRC-dCNN were less accurate than both known-LNBRC-dCNN and joint-LNBRC-dCNN, and further decreased with increasing eye movements. Error bars in all panels correspond to the standard deviation of the sample mean. **(b)** Eye position estimation error as a function of the magnitude of movement, for the same experimental preparations. When eye movements were ignored (zero-LNBRC-dCNN, dotted line), the error in estimated eye position increased linearly, as expected with a 2D Brownian motion. When eye movements were jointly estimated (joint-LNBRC-dCNN; solid lines), the error increased, but more gradually. **(c)** Parasol-only (solid line) and midget-only (dashed line) joint-LNBRC-dCNN image reconstruction performance as a function of the magnitude of movement, for the same experimental preparations. In all preparations, reconstruction quality increased with eye position jitter for both parasol-only and midget-only reconstructions. Midget-only reconstructions had systematically better quality than parasol-only reconstructions in all preparations. **(d)** Parasol-only (solid line) and midget-only (dashed line) eye position estimation error, for the same experimental preparations. For both parasol-only and midget-only reconstructions, the eye position estimation error increased more slowly than if eye movements were ignored (dotted line).

To test whether jitter eye movements improve or degrade retinal coding of natural images, reconstruction quality was examined as a function of eye jitter magnitude. In all three preparations, when simultaneously estimating both the image and eye positions, the mean reconstructed image quality increased with the magnitude of jitter over nearly the entire naturalistic range tested (Fig. 3a, solid). The same was true when reconstructing with known eye positions (Fig. 3a, dashed), demonstrating that the improvement was due to an improved retinal signal. Validation with the LPIPS perceptual distance measure [Zhang 2018] yielded similar results (Extended Data Fig. 4). Thus, fixational jitter eye movements enhance, rather than degrade, the retinal representation.

The benefits of fixational jitter could in principle arise from an overall increase in spike rates, because RGCs are responsive to intensity changes over time, which are increased in the presence of jitter. Indeed, the mean number of spikes increased with increasing eye movement magnitude: Pearson correlation coefficients were 0.940, 0.988, and 0.884 for three experimental preparations. Thus, at least some of the improvement in reconstructed image quality could be attributed to increased RGC firing.

Image reconstruction could also potentially be improved by more accurate estimation of the jitter trajectory with larger eye movements. This did not appear to be the case: the accuracy of eye trajectory reconstruction declined with increasing magnitude of eye position jitter, albeit much more slowly than for the model that assumed zero movement (Fig. 3b). Thus, the improved image reconstruction with increasing magnitude of eye movements was attributable to a more faithful encoding of the stimulus in RGC spikes rather than a more precise implicit signal about eye position.

The potentially distinct impacts of fixational jitter eye movements on each of the parasol and midget RGC representations of the stimulus were examined by reconstructing with one population at a time. Midget-only reconstructions had systematically higher quality than parasol-only reconstructions and contained greater fine spatial detail (Fig. 3c, also Extended Data Fig. 5), demonstrating that midget cells encoded a greater fraction of the stimulus than parasol cells. Reconstruction quality improved with increasing jitter magnitude for both the parasol-only and midget-only reconstructions, demonstrating that jitter eye movements tended to improve representations of the stimulus in both populations. Also, for both populations, the error in estimated eye position increased much more slowly than if eye movements were ignored (Fig. 3d), showing that both cell groups were informative of the eye movement trajectory. However, the position error was substantially smaller in the midget-only case, suggesting that the midget RGCs were largely responsible for encoding fine eye movements.

### Fixational eye movements evoke more precisely timed spikes

**Figure 4.**
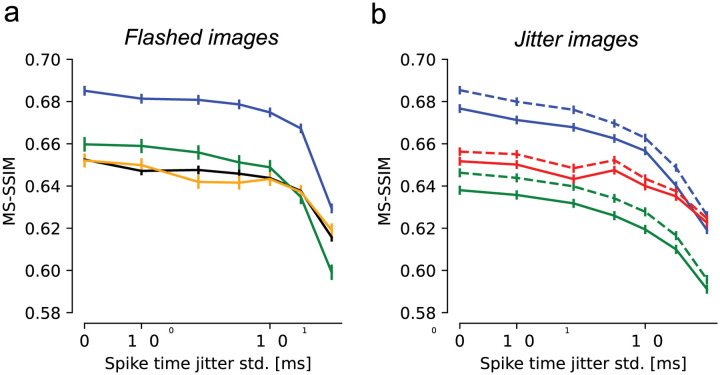
Effects of spike timing precision on reconstruction quality. **(a)** Reconstruction performance for flashed images as a function of spike timing perturbation, in four experimental preparations (colors). Error bars in all panels correspond to the standard deviation of the sample mean. Reconstruction degraded modestly up to spike time perturbations of ~10 ms. **(b)** Reconstruction performance for jittered images. Blue and green lines correspond to the same-colored preparations in (a). Dashed lines correspond to estimation with known eye movement trajectories, solid lines to joint estimation of the image and eye trajectory. In both cases, performance declined smoothly starting at a jitter of ~2–5 ms.

Previous work in the turtle retina has revealed greater temporal precision of RGC spikes in the presence of simulated fixational eye movements [Greschner 2002]. To test whether this could enhance natural image reconstruction, the observed RGC spikes were randomly perturbed in time according to Gaussian distributions with increasing standard deviation (0, 1, 2, 5, 10, 20, and 40 ms), and reconstruction was performed with the perturbed spikes. To ensure optimal reconstruction with the perturbed spikes, the LNBRC models used for estimating likelihood were refitted to perturbed data. Spike time perturbation had two effects on the retinal signal. First, it disrupted the spike train temporal structure, resulting in reduced strength of the fitted LNBRC feedback filter (not shown). Second, because the spike times of each cell were shifted independently, it reduced the spiking synchrony between neighboring cells, resulting in reduced strengths of the LNBRC coupling filters (not shown). For the flashed stimuli, reconstruction quality declined slowly with spike time perturbations up to about 10 ms, and then declined more sharply for larger perturbations, indicating that spike time structure finer than 10 ms was relatively unimportant (Fig. 4a). However, for jittered stimuli, reconstruction quality deteriorated more rapidly as a function of spike time perturbation, and was affected more than the flashed reconstructions by perturbations on the order of 5 ms (see Discussion). This was true regardless of whether eye movements were jointly estimated (Fig. 4b, solid lines) or known *a priori* (Fig. 4b, dashed lines). Repeating the analysis with the LPIPS perceptual distance measure yielded similar results (Extended Data Fig. S7). Thus, eye movements encode the spatial structure of natural images into the fine temporal structure of spikes, and exploiting this encoding enhances decoding.

### Correlated firing between RGCs contributes to reconstructed image quality

Although previous work [Pillow 2008, Ruda 2020] has demonstrated that correlated firing of RGCs affects the visual information transmitted by the retina for simple stimuli, the importance of such correlations in retinal representations of naturalistic stimuli is less certain [Nirenberg 2001, Meytlis 2012, Schneidman 2003], as are the distinct roles of stimulus-dependent (signal) and stimulus-independent (noise) correlations. To better understand the role of correlations in visual signaling by the retina, natural image reconstruction was performed with a readout that ignored all correlations, or with data shuffled to eliminate noise correlations.

**Figure 5:**
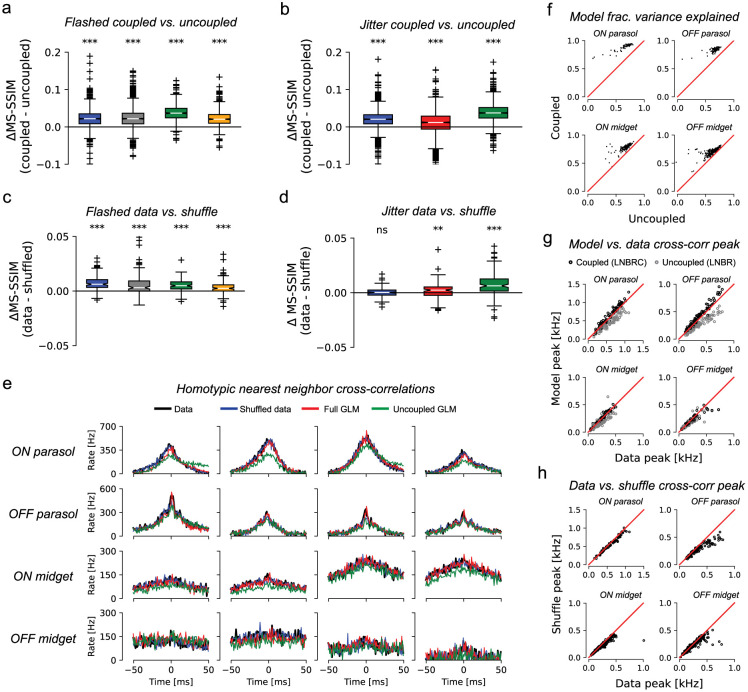
Effects of coupling. **(a)** Differences in reconstruction quality between the coupled model (LNBRC) and uncoupled model (LNBR) for flashed natural images. Mean differences for four preparations (left to right): 0.023, 0.024, 0.037, and 0.023 (all p-values < 1·10^−10^, Wilcoxon one-sided ranked sign test). For all boxplots (panels a-d), the box marks the median and the inter-quartile range (IQR), while the whiskers extend to 1.5 times the IQR. Outliers are marked with a +. **(b)** Same as (a), for jittered image reconstruction, using the joint approach. Mean differences for three preparations (left to right) of 0.020, 0.010, and 0.038 (all p-values < 1·10^−10^). The blue and green boxes in (b) correspond to the same experimental preparations as the blue and green boxes in (a). **(c)** Differences in reconstruction quality between the unshuffled and shuffled trials for flashed image reconstructions, using LNBRCs fitted to unshuffled data, for the same four preparations as (a). Mean differences (left to right): 7.4·10^−3^, 6.6·10^−3^, 5.1·10^−3^, and 3.4·10^−3^ (all p-values < 1·10^−10^). While significant, these differences were substantially smaller than those in (a). **(d)** Differences in reconstruction quality between unshuffled and shuffled trials for jittered image reconstructions, using LNBRCs fitted to unshuffled data, for the same three preparations as (b). Mean differences (left-to-right): 1.5·10^−4^, 1.5·10^−3^, and 7.8·10^−3^ (p-values 0.35, 1.4·10^−3^, and 2.3·10^−14^). The differences were substantially smaller than those in (b). **(e)** Example homotypic (same cell type) nearest-neighbor spike train cross correlograms, computed for the blue experimental preparation from panels (a-d) using repeat presentations of jittered natural image stimuli. Cross-correlograms for the data are shown in black, and for repeat-shuffled data in red. Simulated cross-correlograms for the LNBRC (coupled) models and for the LNBR (uncoupled) models are shown in red and green, respectively. Cross-correlograms computed using the flashed natural image stimulus were similar. The cross-correlograms for unshuffled data, trial-shuffled data, and LNBRC-simulated spike trains were similar, but the LNBR-simulated cross-correlograms did not match the data. **(f)** Comparison of the fraction of PSTH variance explained by the coupled and uncoupled LNBRCs using the jittered stimuli, for the same preparation as in (e). For each of the major cell types, for nearly every cell, the LNBRCs explained a greater fraction of the PSTH variance than the LNBRs. The same comparison was made for the flashed stimulus, with similar results (not shown). **(g)** Comparison of LNBRC-simulated cross-correlogram peak height with the peak height from data, a measure of the degree to which the encoding models accurately represent correlated firing using the jittered stimulus. While the LNBRCs sometimes overestimated the correlations in the data, the LNBRs systematically underestimated them. **(h)** Comparison of the cross-correlogram peak height for repeat data and shuffled repeat data, for the same preparation as (f-g), with the jittered stimulus. With the exception of the OFF parasol cells, peak heights were similar for the data and shuffled cross-correlograms, indicating that noise correlations were only weakly present. The flashed stimuli yielded similar results (not shown).

To probe the overall role of correlations, LNBR (“uncoupled”) encoding models were fitted to the experimental data, and the resulting natural image reconstructions were compared to the results obtained with the full LNBRC (“coupled”) model, similar to previous analyses for white noise stimuli [Pillow 2008]. The uncoupled models lacked the ability to represent correlated firing between RGCs beyond linear filtering of the shared visual stimulus, and were fitted and used to compute reconstructions in an identical manner to the coupled models. For both the flashed and jittered stimuli, the reconstructions computed using the coupled models were significantly more accurate than those computed using the uncoupled models. For the flashed stimuli (Fig. 5a), the mean MS-SSIM differences between coupled and uncoupled reconstructions were 0.023, 0.024, 0.037, and 0.023, (p-values < 1·10^−10^, coupled > uncoupled, Wilcoxon signed rank test, N=1500, N=1750, N=750, and N=750, respectively), and for the jittered stimuli (Fig. 5b) the differences were 0.020, 0.010, and 0.038 (p-values < 1·10^−10^, coupled > uncoupled, Wilcoxan signed rank test, N=1992 for all). Thus, for naturalistic stimuli, knowledge of correlated firing properties of RGCs beyond that which could be explained by linear filtering of the shared stimulus was necessary to effectively decode image content.

The impact of correlated firing on natural image reconstruction could not be attributed to noise correlations alone, in contrast to what was seen in prior work using white noise stimuli [Pillow 2008]. While the cross-correlograms simulated with the coupled LNBRC model (Fig. 5e, red) accurately matched both real data (black) and data shuffled across repeats to remove noise correlations (blue), the cross-correlograms simulated with the uncoupled LNBR model (green) often differed markedly from both. This indicates that the coupled model better represented signal correlations in RGC firing than the uncoupled model. The coupled model also explained a systematically greater fraction of firing variation than the uncoupled model (Fig. 5f).

To probe whether noise correlations contributed significantly to the retinal signal, images were reconstructed from synthetic data created by shuffling the recorded responses of each cell across repeated presentations of the same stimulus. Shuffling removed trial-specific noise correlations between cells, but preserved the firing properties of each cell and stimulus-driven signal correlations between cells. Using the LNBRC fitted to the unshuffled data (i.e. with full knowledge of noise correlations), reconstructions were obtained for both the real (unshuffled) repeats as well as the shuffled data. For the flashed stimuli, the reconstructions computed from unshuffled spikes were marginally more accurate than those computed from the shuffled spikes, for all preparations tested, with mean difference values of 7.4·10^−3^, 6.6·10^−3^, 5.1·10^−3^, and 3.4·10^−3^ (p-values < 1·10^−10^, data > shuffled, Wilcoxon signed rank test, N=150 for all) respectively. For the jittered stimuli, the effect was similar: the difference was significant for two of the three preparations tested, with mean values 1.5·10^−4^, 1.5·10^−3^, and 7.8·10^−3^, (p-values 0.35, 1.4·10^−3^, and 2.3·10^−14^, data > shuffled, Wilcoxan signed rank test, N=149 for all). While statistically significant, the effect was substantially smaller than that of removing the coupling filters, suggesting that the contributions of noise correlations to the retinal representation of natural stimuli were modest. Analysis using the LPIPS perceptual distance measure yielded similar results (Extended Data Fig. 7). Furthermore, comparison of the raw and shuffled repeat cross-correlograms (black and blue lines in Fig. 5e for data and shuffled, respectively) and cross-correlogram peak height (Fig. 5h) showed that noise correlations were substantially smaller than signal correlations. These striking differences compared to reconstruction performed previously using white noise stimuli [Pillow 2008] highlight the importance of understanding visual encoding of naturalistic scenes with eye movements.

## Discussion

We have presented a Bayesian method to invert the retinal code, reconstructing visual images from the spiking responses of a population of RGCs. This reconstruction is not intended as a description of how the brain processes visual images [Dennett 1992], but as a tool for making explicit the content of the retinal signal in the form of an image, providing insight into the sensory content that is available in neural activity and the way this content is represented [Rieke 1997].

These analyses relied on both the performance and interpretability of the reconstruction method, leveraging both the sophistication of and separation between the likelihood and prior models. The likelihood, obtained from an LNBRC encoding model, effectively captured RGC responses to naturalistic stimuli with modular components that represented stimulus dependency, spike history dependence, and spike time correlations. Although it is not matched to the details of biological circuitry or cellular biophysics [Trong 2008, Vidne 2012], it is convex in its parameters, and thus reliably fit to spiking data and computationally feasible to use for the MAP reconstruction problem. Separately, natural image structure was captured using the prior implicit in a neural network trained to denoise images. Such implicit priors, related to the “score-based generative models” or “diffusion models” that have recently emerged in the machine learning community, offer unprecedented power for capturing image properties while requiring relatively modest amounts of training data. Most importantly, the likelihood and prior components together provide a Bayesian formulation, which offers enhanced interpretability because the components can be independently altered to evaluate their contributions to the retinal representation.

The reconstruction approach reveals that the retinal signal alone is sufficient for accurately decoding visual stimuli in the presence of unknown fixational eye movements, consistent with previous theories [Gibson 1954, Pitkow 2007, Burak 2010, Anderson 2020] and psychophysical studies [Murakami 1998, Poletti 2010, Arathorn 2013, Ratnam 2017]. Though previous computational investigations [Pitkow 2007, Burak 2010, Anderson 2020] have explored this possibility in simulation with simplified stimuli, the present work tested it empirically with efficient reconstruction of naturalistic stimuli using recorded RGC responses. Of course, the present findings do not exclude the possibility of additional extra-retinal signals that could help to compensate for fixational eye movements, as has been reported previously [Zhang 2023]. Indeed, the small gap in quality between images reconstructed by the joint algorithm and those reconstructed with full knowledge of the eye position suggests possible benefits of incorporating extra-retinal signals.

Increased fixational jitter was found to improve reconstruction quality. This provides additional evidence in support of the theory that fixational eye movements serve a useful function in visual processing, modulating high frequency spatial detail into time domain [Rucci 2007, Kuang 2012, Rucci 2015, Boi 2017] and/or enabling super-resolution imaging [Ratnam 2017, Anderson 2020]. Furthermore, because this finding held even when the eye movements were unknown *a priori*, it demonstrates that jitter eye movements specifically improve the fidelity of the retinal representation of natural images.

Precisely timed spikes were shown to play an important role in the retinal representation of jitter eye movements. Though RGCs can spike with temporal precision on the order of 1 ms [Berry 1997, Reich 1997, Keat 2001, Uzzell 2004], previous studies have shown that longer integration times (~10 ms) provide the highest-fidelity readout of steady visual motion from RGCs [Chichilnisky 2003, Frechette 2005]. Consistent with these studies, and with previous flashed natural image reconstruction [Kim 2020, Brackbill 2020], we found that flashed image reconstruction was robust to spike train temporal perturbations up to 10 ms. However, in the presence of jitter eye movements, finer temporal precision (2–5 ms) was required for optimal reconstruction. This is consistent with work suggesting that the spike train temporal structure induced by fixational eye movements encodes high-frequency spatial detail [Greschner 2002, Poletti 2008, Kuang 2012].

As in previous work on reconstruction of white noise stimuli [Pillow 2008, Ruda 2020], correlated RGC firing was critical for reconstructing jittered natural images (but see [Meytlis 2012]). Surprisingly, however, the effect for naturalistic stimuli was primarily attributable to stimulus-driven correlations rather than the noise correlations that dominated the results in the prior work. The weak role of noise correlations in the present data matched the results obtained by reconstructing flashed natural images using more limited approaches [Brackbill 2020, Kim 2020] and results from decoding dynamically-varying artificial movies [Botella-Soler 2018].

Future work could extend the Bayesian reconstruction framework to characterize the function of spatio-temporal nonlinearities in the retinal representation of naturalistic stimuli. Though recent work with subunit [Freeman 2015, Liu 2017, Shah 2019] and neural network [McIntosh 2016] encoding models has demonstrated substantial improvements in accounting for RGC spiking, the roles of the spatio-temporal nonlinearities contained in these models for visual signaling remain unclear. Combining such encoding models with denoising image priors to draw samples from the posterior [Kadkhodaie 2021, Chung 2023, Zhu 2023] could more deeply probe the interplay between retinal coding and natural image statistics.

## Methods

### Multi-electrode array recordings

Large-scale electrophysiological recordings from *ex vivo* macaque monkey retina were performed using a 512-electrode multi-electrode array system [Litke 2004] with 60 μm pitch between electrodes and a 2×1 mm rectangular recording area. The retinas were obtained from terminally-anesthetized macaques used by other laboratories, in accordance with Institutional Animal Care and Use Committee requirements. A complete description of the experimental procedures can be found in Brackbill *et al.* [Brackbill 2020].

Spike sorting was performed with YASS [Lee 2020]. RGCs of the four numerically dominant types in macaque (ON parasol, OFF parasol, ON midget, OFF midget) were identified manually based on receptive fields and autocorrelation functions characterized with a spatio-temporal white noise stimulus according to previously described procedures [Rhoades 2019], and were matched to spike-sorted units from the natural scenes recordings by matching electrical images (voltage templates). Only identified RGCs of the four major cell types were used in the analysis. The four preparations used for the flashed reconstructions contained 691, 592, 704, and 677 total cells, and the three preparations used for the jitter eye movements reconstructions contained 715, 604, and 775 total cells.

### Visual stimulus

Flashed natural images from the ImageNet database were presented to the retina according to [Brackbill 2020]. Images were converted to grayscale, cropped to 256×160 resolution, and padded with gray borders. The stimulus extended beyond the boundaries of retinal preparation and fully covered all receptive fields. Each pixel in the image measured approximately 11 × 11 μm when projected on the retina. Each image was displayed for 100 ms (12 frames at 120 Hz), and sequential images were separated by a 400 ms uniform gray screen. Additional details for the visual stimulus and setup are provided in Brackbill *et al*. [Brackbill 2020].

The natural movies with simulated fixational eye movements consisted of ImageNet images presented for 500 ms each (60 frames at 120 Hz), with no gray screen separation. For each image, eye movements were simulated by shifting the image during each frame transition according to a discretized 2D Brownian motion with diffusion constant of 10 μm^2^/frame, consistent with estimates of fixational eye movements in both human [Kuang 2012, Rucci 2015] and non-human primate [Z.M. Hafed and R.J. Krauzlis, personal communication, June 2008]. Simulated eye movements were drawn independently of the image. The movies were presented in sequence, with no gray screen between movies.

The receptive fields of the recorded RGCs covered only a central region of the stimulus field, leaving a perimeter region for which no cells were recorded. To evaluate image quality only over regions of the stimulus corresponding to recorded cells, a valid region was constructed, consisting of the convex hull of the receptive fields of the full RGC population. Only pixels in this valid region were used to compute image quality.

### Fitting LNBRC models of RGC spiking

The linear-nonlinear-Bernoulli with recursive coupling (LNBRC) is a modified form of the model presented in [Pillow 2008]. It generalizes the classical linear-nonlinear-Poisson (LNP) spiking model by incorporating recursive feedback (spike history) and neighboring cell coupling filters to model spike train temporal structure and cell-to-cell correlations (Fig 1b). For RGC *i*, the LNBRC has the following parameters: (1) **m**_*i*_, the linear spatio-temporal stimulus filter; (2) *f*_*i*_[*t*], the recursive feedback filter; (3) $c_{i}^{\left(j\right)}[t]$, the coupling filters to neighboring RGCs indexed by *j*. where neighboring cells were included if their receptive field centers fell within twice the median nearest neighbor distance for parasol cells and 2.5 times the median nearest neighbor distance for midget cells; and (4) *b*_*i*_, an additive bias. Let **v**[*t*] denote a temporal window of the visual stimulus movie up to and including time *t*, * a time-domain convolution, and *s*_*i*_ the spike train of cell *i*. The instantaneous spiking probability for cell is computed from the *generator signal*, *g*_*i*_ [*t*]:

$$g_{i}[t]=m_{i}^{T}(v[t-1])+\left(s_{i}*f_{i}\right)[t-1]+\sum_{j\in \text{neighbors}}\left(s_{j}*c_{i}^{\left(j\right)}\right)[t-1]+b_{i}.$$

All filters in the LNBRCs were strictly causal, so that the firing probability at time *t* depended only on the visual stimulus and observed spikes occurring strictly before time *t*. Time was discretized in 1 ms bins, corresponding approximately to the duration of the refractory period of a neuron. Since at most one spike could occur in each time bin, a Bernoulli random process was used to model spiking, with a sigmoidal nonlinearity of the form $\frac{e^{x}}{1+e^{x}}$ mapping the generator signal to an instantaneous firing probability, resulting in the encoding negative log-likelihood

$$-\text{log}\;p(s|v)=\sum_{t}\left[\text{log}\left(1+\text{exp}\left\{g_{i}[t]\right\}\right)-s_{i}[t]g_{i}[t]\right],$$

which is jointly convex in the model parameters. The stimulus filter was assumed to be space-time separable (rank 1), and the stimulus filter spatial component was additionally cropped to a rectangular region surrounding the cell’s receptive field and represented in terms of a 2D cubic spline basis [Huang 2021]. The feedback, coupling, and time component of the stimulus filter were each parameterized as linear combinations of low-rank 1D raised cosine basis functions [Pillow 2008].

The models were fitted to recorded RGC spikes by maximizing the parameter likelihood, and were regularized with an L_1_ penalty to induce sparsity on the spatial component of the stimulus filter, and an L_2,1_ group-sparsity penalty on the cosine basis representation of the coupling filters to eliminate spurious cell-to-cell correlations. Because of the assumed space-time separability of the stimulus filter, the LNBRCs were fitted using coordinate descent, alternating between solving a spatial convex minimization problem in terms of the stimulus spatial filter, feedback filter, coupling filters, and bias, and solving a temporal convex minimization problem in terms of the stimulus time course filter, feedback filter, coupling filters, and bias. All optimization problems were solved using FISTA, an accelerated proximal gradient method [Beck 2009], using the formulation for the L_2,1_-regularized problem presented in [Liu 2009]. Optimal values for the weights placed on the L_1_ and L_2,1_ regularizers were found using a grid search to minimize the mean test negative log-likelihood over four randomly chosen cells of each cell type. Within each preparation, every RGC of a given type used the same hyperparameters.

The LNBRCs were fitted separately for each cell, and required about 180 seconds of compute time per cell for the static stimulus models and 500 seconds of compute time per cell for the eye movements models on a single NVIDIA V100 GPU with 32 GB of VRAM.

### LNBRC simulated spike train generation

Simulated spike trains for evaluating model fit quality were generated from the LNBRC by computing the value of the generator signal from the stimulus and using simulated Bernoulli random variables to model random spike generation. The recursive feedback contribution to the generator signal was initialized using real observed spike trains, and subsequent generated spike trains were fed back into the model to compute the feedback contribution for future spikes. Because the firing probability computed with the coupled LNBRC was conditional not only on the visual stimulus and simulated cell spiking history, but also on the spike trains of nearby coupled RGCs, real spike trains from the experimental data were used to compute the coupling contribution to the generator signal.

### PSTH computation

The peri-stimulus time histogram (PSTH) was computed using RGC responses to repeat presentations of the same visual stimulus, by binning the observed spikes into time bins with 1 ms width, smoothing with a Gaussian kernel with standard deviation of 2 ms, and then computing the mean over all repeated presentations of the stimulus.

### Fitting benchmark LNP encoding models

Benchmark linear-nonlinear-Poisson (LNP) encoding models were fitted in a similar manner to the LNBRCs. The same spatial basis sets used for the LNBRCs were used for the LNP models. Spikes trains were binned into counts with 8.33 ms time bins, corresponding to one bin per stimulus frame. LNP models were parameterized by a spatio-temporal stimulus filter **m**_*i*_, and a bias *b*_*i*_, resulting in a generator signal of the form $g_{i}[t]=m_{i}^{T}(v[t])+b_{i}$. An exponential nonlinearity was used, resulting in a encoding negative log-likelihood with form

$$-\text{log}\;p(s|v)=\sum_{t}\left[\text{exp}\;g_{i}[t]-g_{i}[t]s_{i}[t]\right]$$

which is convex in the LNP model parameters. LNP spatio-temporal filters were assumed to be rank-1 space-time separable. An L_1_ penalty was used to induce sparsity in the spatial component of the stimulus filter, and the corresponding weight for that penalty was chosen by performing a grid search with encoding likelihood on the test partition as the objective. Models for each cell were fitted using FISTA.

### Reconstruction of flashed images with denoising CNN prior

An iterative Plug-and-Play algorithm [Venkatakrishnan 2013, Teodoro 2019, Zhang 2021] was used to perform MAP reconstruction of flashed static natural images. Rather than solve the MAP problem directly, the algorithm used proximal variable splitting to divide the MAP objective $\text{arg}\;\underset{y}{\text{min}}\{-\text{log}\;p(s|y)-\lambda \;\text{log}\;p(y)\}$ into an encoding sub-problem $x^{\left(k+1\right)}=\text{arg}\;\underset{x}{\text{min}}\left\{-\text{log}\;p(s|x)+\frac{\rho^{\left(k\right)}}{2}{\left|x-z^{\left(k\right)}\right|}_{2}^{2}\right\}$ and a prior sub-problem $z^{\left(k+1\right)}=\text{arg}\;\underset{{}_{z}}{\text{min}}\left\{-\lambda \;\text{log}\;p(z)+\frac{\rho^{\left(k\right)}}{2}{\left|z-x^{\left(k+1\right)}\right|}_{2}^{2}\right\}$ and iteratively alternated between the two. The encoding sub-problem was solved using unconstrained convex minimization. The prior sub-problem has the form of a MAP estimation problem for images contaminated with additive Gaussian noise. As such, its solution was approximated using a single forward pass of a convolutional neural network (CNN) pretrained for denoising with specified noise variance $\frac{\lambda }{\rho^{\left(k\right)}}$. Ten iterations of alternating optimization were used. ρ^(*k*)^ was increased per iteration on a log-spaced schedule [Zhang 2021], and hyperparameters λ, ρ^(1)^ and ρ^(10)^ were found by performing a grid search on an 80-image subset of the test partition with reconstruction MS-SSIM as the objective. A detailed description of the algorithm can be found in [Wu 2023].

### Exact MAP reconstruction with 1/F Gaussian prior

Using the 1/F Gaussian prior, the MAP objective had the form

$$\text{arg }\underset{{}_{y}}{\text{min}}\left\{-\text{log}\;p(s|y)+\lambda \sum_{k}{\left|a_{k}(y)\right|}^{2}/f_{k}^{2}\right\}$$

where *a*_*k*_(*y*) is the amplitude of the Fourier coefficient at frequency *f*_*k*_. Because both the 1/F prior term and the encoding negative log-likelihood are smooth and convex in the image, the MAP problem is an unconstrained convex minimization problem and hence was solved with gradient descent. The optimal value of the prior weight λ was found with a grid search with reconstruction MS-SSIM as the objective.

### Approximate MAP reconstruction with known eye movements with denoising CNN prior

In the case that the eye movements *w* are known *a priori*, the MAP objective can be simplified into the form

$$\hat{y}=\text{arg }\underset{{}_{y}}{\text{min}}\{\text{log}\;p(s|y,w)+\lambda \;\text{log}\;p(y)+\text{log}\;p(w)\}\;=\text{arg }\underset{{}_{y}}{\text{min}}\{\text{log}\;p(s|y)+\lambda \;\text{log}\;p(y)\}$$

which can be solved using the Plug-and-Play algorithm described above for the flashed case. Hyperparameters were found with a grid search with MS-SSIM as the objective.

### Joint estimation of image and unknown eye movements with denoising CNN prior

The expectation-maximization (EM) algorithm was used to perform MAP estimation for joint estimation of images and eye movements. Letting *w* denote the eye movement trajectory over all timesteps, the exact MAP problem with unknown eye movements has form

$$\text{arg }\;\underset{{}_{y}}{\text{max}}\{\lambda \;\text{log}\;p(y)+\text{log}\sum_{w}p(s|y,w)p(w)$$

which cannot be directly solved because the marginalization over all possible eye movement trajectories *w* is intractable. MAP-EM offers an iterative approach for estimating the image *y*, and consists of alternating steps of: (1) finding the image that maximizes the sum of the evidence lower bound and natural image log prior

$$y^{\left(i\right)}=\text{arg}\;\underset{y}{\text{max}}\left\{\lambda \;\text{log}\;p(y)+E_{w\sim q\left(w|y^{\left(i-1\right)},s\right)}[\text{log}\;p(s|y,w)]\right\}$$

over some variational distribution of the eye positions *q*(**w** | y^(*i*−1)^, *s*); and (2) using the resulting estimate of the image *y*^(*i*)^ to update the variational distribution. For computational tractability, we assumed *q* had form $q\propto p(s|w,y)r_{0}\left(w_{0}\right)\prod_{i=1}^{T}r_{i}\left(w_{i}|w_{i-1}\right)$, where *r* could be an arbitrarily chosen distribution. *q* was represented approximately using a weighted particle filter with N=10 particles. The particle filter was updated once for each frame transition (every 8.33 ms) using a sequential importance resampling procedure [Liu 1998]. Specifically, at frame *t*, the trajectory represented by each particle was updated by sampling a new eye position from the 2D Gaussian transition probability distribution *p*(**w**_*t*_ | **w**_*t*−1_), and then reweighting each particle using the multiplicative weight $\frac{p\left(s|w^{\left(t\right)},y\right)}{p\left(s|w^{\left(t-1\right)},y\right)}$ computed using the encoding likelihood model. Mathematical details for the resampling particle filter, including justification for the weight update rule, are provided in the Supplement.

An initial guess for the image *y*^(0)^ was reconstructed by assuming fixed eye position at the origin and performing ten alternating iterations of the algorithm used for the flashed reconstructions. At each intermediate timestep *i*, updated estimates of the image *y*^(*i*)^ were computed by performing a single encoding optimization step $x^{\left(i\right)}=\text{arg}\;\underset{{}_{x}}{\text{min}}\left\{-E_{w\sim q\left(w|y^{\left(i-1\right)},s\right)}[\text{log}\;p(s|x,w)]+\frac{\rho^{\left(i\right)}}{2}{\left|x-y^{\left(i-1\right)}\right|}_{2}^{2}\right\}$ using unconstrained convex minimization, followed by a single prior optimization step $y^{\left(i\right)}=\text{arg}\;\underset{z}{\text{min}}\left\{-\lambda \;\text{log}\;p(z)+\frac{\rho^{\left(i\right)}}{2}{\left|z-x^{\left(i\right)}\right|}_{2}^{2}\right\}$ using a single forward pass of the Gaussian denoiser. To speed computation, images were updated once for every five display frame transitions. Testing on a subset of data indicated that this did not negatively affect reconstruction quality.

### Reconstruction quality evaluation

Reconstruction quality was evaluated using Multi-scale Structural Similarity (MS-SSIM) [Wang 2004], a widely used metric for perceptual similarity. MS-SSIM was calculated over the valid region of the image (described above), ignoring non-informative regions of the stimulus for which no RGCs were recorded. For the jittered reconstructions, the absolute position of the reconstructed image was arbitrary (having been jointly estimated from many jittered input samples), and MS-SSIM was computed for a range of pixel-wise shifts of the reconstructed image, and the best value over all shifts was used.

The results in the paper were also confirmed using the Learned Perceptual Image Patch Similarity (LPIPS) [Zhang 2018], an alternative measure of perceptual distance computed using pre-trained neural network classifiers. LPIPS has different working principles than MS-SSIM, and has been shown to align with human perceptual judgements. Only pixels within the valid region (described above) were used to compute LPIPS.

### Cross-validation data rotation for eye movements analysis

Five-fold data rotation was used to maximize the number of stimulus images available for determining the effect of jitter eye movements on reconstructed image quality. Five different sets of LNBRCs were fitted, each corresponding to distinct and non-overlapping test and held out partitions, such that test-quality reconstructions could be produced for nearly every stimulus image presentation in the recorded dataset.

### Cell-type-specific reconstruction analysis

The cell-type-specific analysis was performed by reconstructing the jittered eye movements stimulus using joint-LNBRC-dCNN. For simplicity, the LNBRC models used for this analysis only modeled homotypic correlations, differing from the models used elsewhere in the work. Five-fold data rotation was used for this analysis.

### Spike time perturbation analysis

The spike time perturbation analysis tested the temporal precision of the retinal code by shifting recorded spike times by random amounts drawn from a zero-mean Gaussian, with standard deviations of 1 ms, 2 ms, 5 ms, 10 ms, 20 ms, and 40 ms. To ensure optimal reconstruction at each level of perturbation, the LNBRCs were refitted to each condition. Images were reconstructed using the time-perturbed data and the time-perturbed LNBRCs using the algorithms described above. Optimal hyperparameters were found separately for each time perturbation condition by performing grid searches.

### Uncoupled (LNBR) model correlations analysis

The LNBR (uncoupled) model removes the neighboring cell coupling filters of the LNBRC model, thus losing the ability to represent correlated firing between nearby RGCs. The LNBR is parameterized by a linear spatio-temporal stimulus filter, a recursive feedback filter, and a bias. Using the same notation as in the fully-coupled case, the generator signal for cell *i* in the uncoupled model is written as

$$g_{i}[t]=m_{i}^{T}(v[t-1])+\left(s_{i}*f_{i}\right)[t-1]+b_{i}.$$

The LNBRs were fitted with the same 1 ms time bins, sigmoidal nonlinearity, and Bernoulli random spiking model as the LNBRCs. Space-time separability of the stimulus filters was assumed, and the same alternating optimization procedure for fitting was used as in the LNBRC case. An L_1_ penalty was used to regularize the spatial component of the stimulus filters, and the optimal value of the corresponding hyperparameter was found using a grid search.

Image reconstruction with LNBRs was done in an identical manner as with the LNBRCs. Reconstruction hyperparameters were found using a grid search.

### Noise correlations shuffled repeats analysis

Noise correlations between RGCs were characterized using responses to repeated presentations of the same stimulus. Shuffled responses were constructed by randomly reordering recorded spike trains for each cell across the repeated trials, eliminating noise correlations while preserving single-cell spiking statistics and stimulus-induced correlations. Images were reconstructed for both the real (unshuffled) trials as well as the shuffled trials using LNBRCs fitted to the unshuffled data, using the reconstruction algorithms described above. The change in reconstructed image quality due to shuffling was then computed by taking the mean reconstruction quality across repeats of the same stimulus, and then subtracting the values computed for the shuffled repeats from the values computed for the data repeats.

### Cross-correlogram computation

Cross-correlograms between cells were computed using repeat stimulus presentations by constructing histograms for the differences in spike times of the cells (with 1 ms bins), and taking the mean over all presentations of the same stimulus. Because the stimulus onset and offset frame transitions in the flashed stimuli and transitions between distinct images for the jittered eye movements stimuli induced simultaneous firing of all cells independent of connectivity and shared input structure, a shift predictor correction to the cross-correlograms was applied [Perkel 1967]. This was done by shifting the spike times for the second cell such that the spike trains for that cell corresponded to the response to a different stimulus image, constructing the histogram for the differences in spike times for the cells, and then subtracting said histogram from the original raw cross-correlogram. This removed the component of the cross-correlogram that could be predicted by the trial structure alone, independent of either the spatial content of the stimulus or of noise correlations.

## Supplementary Material

Supplement 1

## Data Availability

A toy dataset sufficient for generating the example reconstruction images in the figures is provided for review. Upon acceptance at a journal, this will be made publicly available. We are unable to release the raw voltage traces due to their large size (> 5 TB) and the complexity of the data processing pipeline.
