# Supplementary material for: Fixational Eye Movements Enhance the Precision of Visual Information Transmitted by the Primate Retina
Source: bioRxiv. 2023 Sep 5:2023.08.12.552902. Originally published 2023 Aug 14. Preprint. [Version 2] doi: 10.1101/2023.08.12.552902 (PMC10462030; doi:10.1101/2023.08.12.552902)
Supplement: Supplementary file 1 [file media-1.pdf]

Supplemental Information

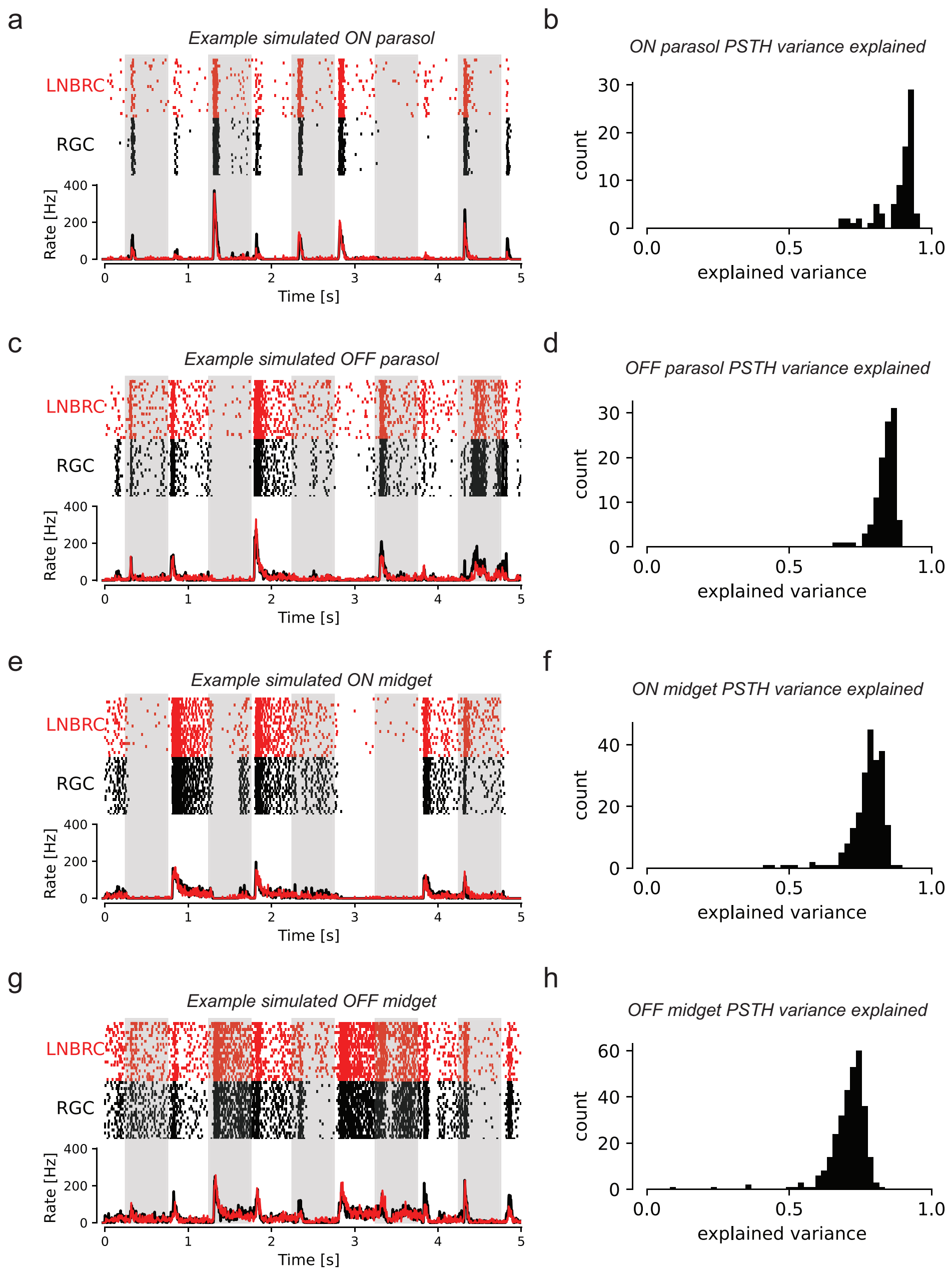

Figure S1. LNBRC fit quality for jittered eye movement natural movies, for cells of each of the major cell types in the macaque monkey (ON parasol, OFF parasol, ON midget, OFF midget). (a) Comparison of LNBRC-simulated repeat rasters and PSTH with real recorded repeats in response to jittered natural movie stimuli, for a single example ON parasol. **(b)** Histogram showing fraction of jittered movie PSTH variance explained by the LNBRCs, for every ON parasol recorded in one preparation. The fraction of explained variance was systematically high for every cell, suggesting that LNBRCs can accurately represent retinal responses to natural movie stimuli. **(c)** and **(d)** Same as (a) and (b) for OFF parasols. **(e)** and **(f)** Same as (a) and (b) for ON midgets. **(g)** and **(h)** Same as (a) and (b) for OFF midgets.

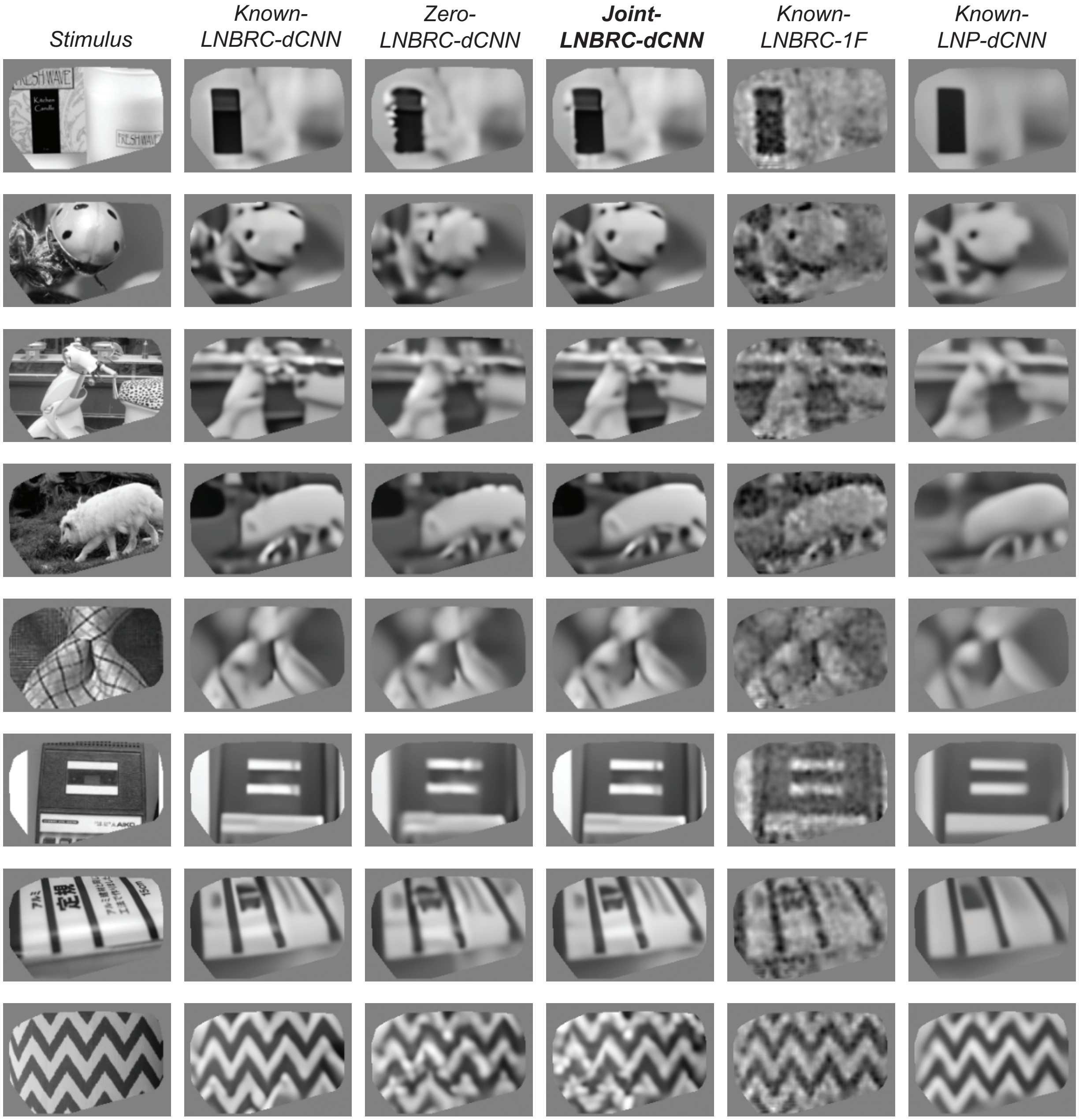

Figure S2: Additional example jitter stimulus images and reconstructions, from the same experimental preparation as Figure 2d from the main text, with additional comparisons against simpler reconstruction methods. Columns (left to right): *Stimulus*, the image presented to the retina; *Known-LNBRC-dCNN*, MAP reconstruction with known eye movements, using LNBRC encoding and dCNN prior; *Zero-LNBRC-dCNN*, MAP reconstruction while incorrectly assuming no eye movements, using LNBRC encoding and dCNN prior; *Joint-LNBRC-dCNN*, reconstruction by jointly estimating image and eye movements, using LNBRC encoding and dCNN prior; *Known-LNBRC-1F*, MAP reconstruction with known eye movements, using LNBRC encoding and simpler 1/F Gaussian prior; *Known-LNP-dCNN*, MAP reconstruction with known eye movements, using simpler LNP encoding and dCNN prior.

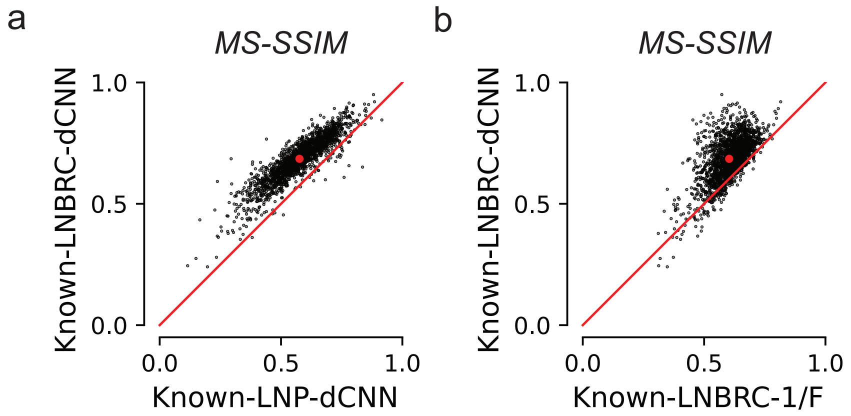

Figure S3: Performance comparison of the LNBRC-dCNN MAP reconstruction algorithm against simpler alternatives, for the eye movements natural movies, in the case that the eye movements are known *a priori*. **(a)** Comparing LNBRC-dCNN against LNP-dCNN, in which the LNBRC encoding model was replaced with a simpler LNP encoding model. Reconstructions using the LNBRC encoding model were systematically better than those using the LNP model, demonstrating that the more sophisticated LNBRC encoding model contributed substantially to reconstruction quality. **(b)** Comparing LNBRC-dCNN against LNBRC-1/F, in which the dCNN prior was replaced with a simpler Gaussian 1/F prior. Reconstructions using the dCNN prior were systematically better than those using the 1/F prior, demonstrating that the dCNN natural image prior contributed substantially to reconstruction quality.

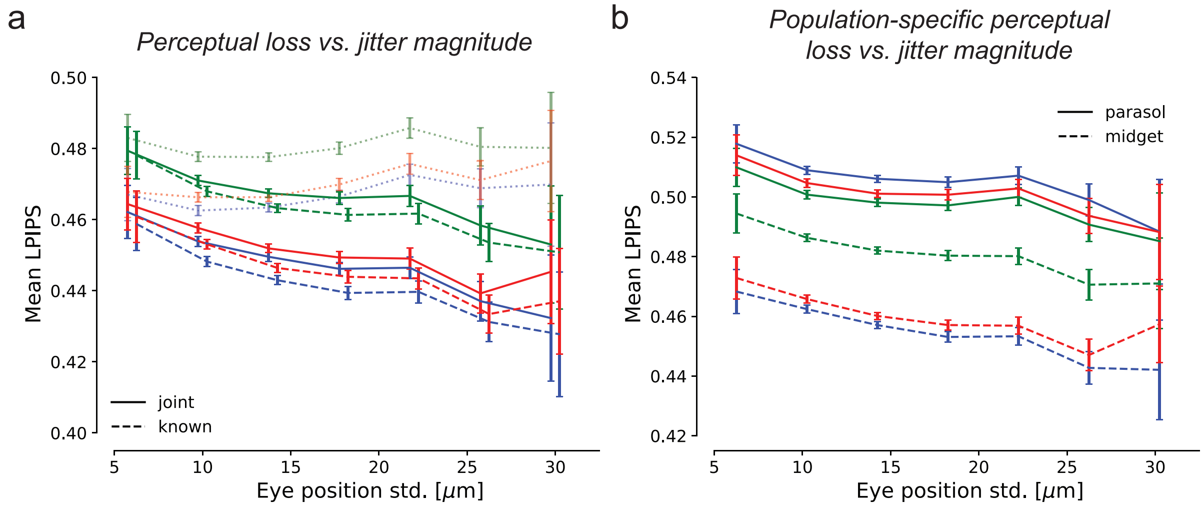

Figure S4: (a) Re-analysis of Figure 3a (reconstruction quality as a function of quantity of jitter eye movements) using LPIPS, an alternative measure of perceptual distance that uses neural networks. Lower LPIPS corresponds to a better reconstruction, and higher LPIPS to a worse reconstruction. Each color corresponds to a different preparation (same color convention as Figure 3a). Error bars correspond to the standard deviation of the sample mean. The results using LPIPS agree with those using MS-SSIM: when compensating for eye movements, regardless of whether eye movements were known *a priori* (Known-LNBRC-dCNN, dashed lines) or jointly estimated along with the image (joint-LNBRC-dCNN, solid lines), LPIPS decreases with increasing eye movements (images improve in quality with increasing eye movements). Failure to compensate for eye movements (zero-LNBRC-dCNN, dotted lines) resulted in LPIPS increasing with increasing eye movements. **(b)** Re-analysis of Figure 3b (population-specific reconstruction quality as a function of quantity of jitter eye movements) using LPIPS. The solid lines correspond to the parasol-only joint-LNBRC-dCNN reconstructions, and the dashed lines to midget-only joint-LNBRC-dCNN reconstructions. Error bars correspond to the standard deviation of the sample mean. In

all cases, LPIPS decreased with increasing eye movements, demonstrating that fixational jitter eye movements improved both the parasol cell and midget cell signals.

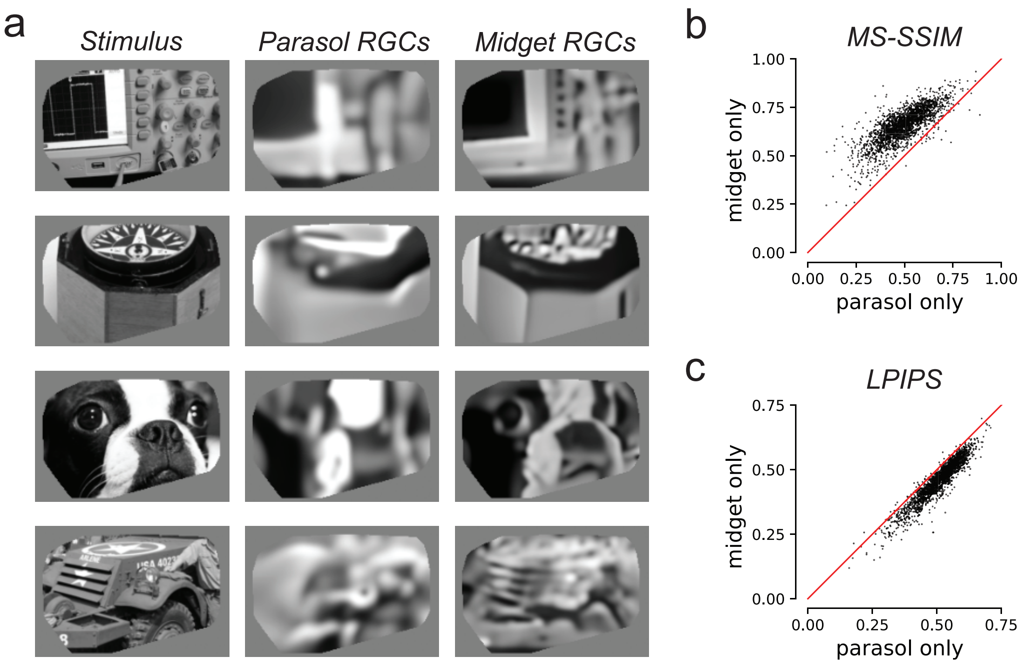

Figure S5: Comparison of parasol-only and midget-only reconstructions for the jitter eye movements stimulus, in one preparation. **(a)** Example reconstructions, using the joint-LNBRC-dCNN simultaneous eye movement estimation and image reconstruction algorithm. Columns (left to right): *Stimulus*, the image presented to the retina; *All RGCs*, reconstructions computed using all parasol and midget RGCs in the preparation; *Parasol RGCs*, reconstructions computed using only parasol RGCs; and *Midget RGCs*, reconstructions computed using only midget RGCs. The midget-only reconstructions contained greater fine spatial detail than the parasol-only reconstructions. **(b)** Comparison of MS-SSIM reconstruction quality between the midget-only and parasol-only reconstructions. For nearly every image, the midget-only reconstruction quality exceeded that of parasol-only reconstructions. **(c)** Comparison of LPIPS perceptual distance between midget-only and parasol-only reconstructions. For nearly every image, the midget-only perceptual distance was smaller than that of parasol-only reconstructions.

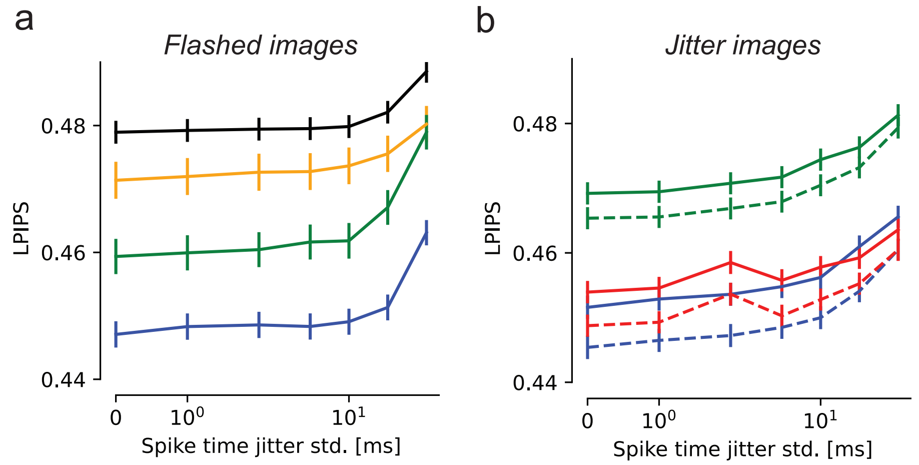

Figure S6: Re-analysis of Figure 4 using LPIPS, a measure of perceptual distance computed using a pre-trained neural network. Because LPIPS measures perceptual distance rather than similarity, smaller values of LPIPS correspond to better reconstructions, while larger values of LPIPS correspond to worse reconstructions. Error bars in all panels correspond to the standard deviation of the sample mean. **(a)** For the flashed image stimulus, LPIPS remained relatively constant up to 10 ms of spike timing jitter. **(b)** For the images jittered to emulate fixational eye movements, LPIPS gradually increased with increasing spike time jitter, and degraded measurably with ~2-5 ms of spike timing jitter.

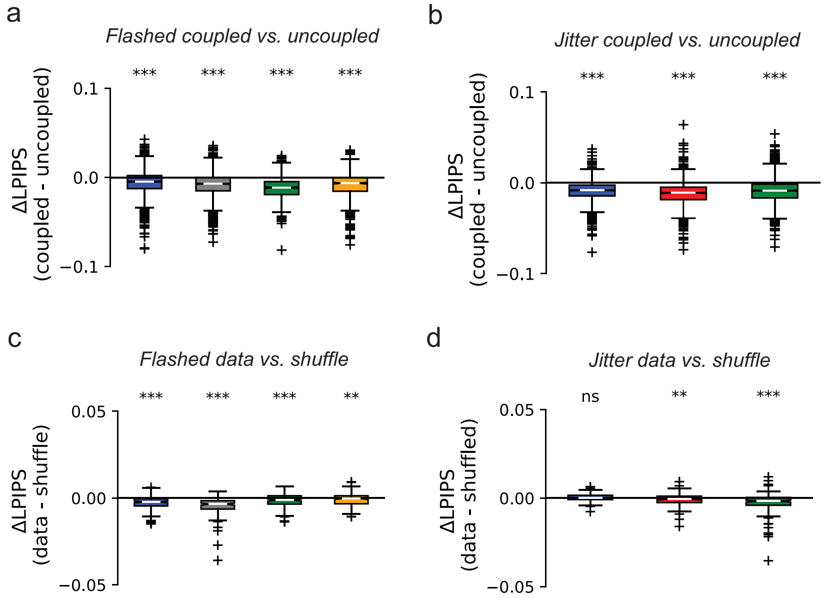

Figure S7: Re-analysis of Figure 5 using LPIPS. (a) For the flashed stimuli, LPIPS distances were significantly lower (better) when using the fully-coupled LNBRC models to perform reconstruction than when using the uncoupled LNBR models (from left to right, mean differences between coupled and uncoupled LPIPS were -5.85·10^{-3}, -8.20·10^{-3}, -1.2·10^{-2}, -8.54·10^{-3}, all p-values < 1·10^{-10}, Wilcoxon signed rank test, coupled < uncoupled, N=1500, N=1750, N=750, and N=750, respectively). For all boxplots (panels a-d), the box marks the median and the inter-quartile range (IQR), while the whiskers extend to 1.5 times the IQR. Outliers are marked with a +. **(b)** The same result held for the jittered stimulus (from left to right, mean differences between coupled and uncoupled LPIPS were -8.69·10^{-3}, -9.02·10^{-3}, and -1.20·10^{-2}, all p-values < 1·10^{-10}, Wilcoxan signed rank test, coupled < uncoupled, N=1992 for all). **(c)** For the flashed stimuli, LPIPS decreased when RGC responses were shuffled across repeats to remove noise correlations, but by a much smaller amount than when the coupling filters were removed (from left to right, mean LPIPS differences between data and shuffled reconstructions were -2.73·10^{-3}, -

$3.48 \cdot 10^{-3}$, $-1.16 \cdot 10^{-3}$, and $-1.85 \cdot 10^{-4}$, with respective p-values $< 1 \cdot 10^{-10}$, $< 1 \cdot 10^{-10}$, $5.45 \cdot 10^{-5}$, and $8.52 \cdot 10^{-3}$, coupled < uncoupled, Wilcoxan signed rank test, N=150 for all). **(d)** The same result held for the jittered stimulus (from left to right, mean difference between data and shuffled reconstructions were $2.50 \cdot 10^{-4}$, $-7.06 \cdot 10^{-4}$, and $-2.55 \cdot 10^{-3}$, with respective p-values 0.946, $2.31 \cdot 10^{-3}$, and $< 1 \cdot 10^{-10}$, coupled < uncoupled, Wilcoxan signed rank test, N=149 for all).

Natural image reconstruction with jittered eye movements using Plug-and-Play MAP-EM

Eric G. Wu

1 Definitions

- The vector $\mathbf{s}$ represents the observed spikes for every cell, for every spiking time bin.
- The jittered movie stimulus (e.g. *with* simulated fixational eye movements) consists of a single static natural image jittered in time, modeling fixational eye movements as 2D Brownian motion. The jitter is discretized pixel-wise and in time, and is parameterized in terms of:
  - $\mathbf{y}$, the static image, a continuous-valued $H \times W$ matrix with each entry corresponding to a pixel value.
  - $\mathbf{w}_{0:T}$, the a $2 \times T$ integer-valued matrix representing the eye movements trajectory, with entries representing the discrete x and y image positions. $\mathbf{w}_{0:T}$ is integer-valued because the simulated eye movements in the jittered movie were generated from a discretized 2D Brownian motion.

  Let $\mathbf{w}_t$ represent the shift at timestep t. Frame t of the jittered movie stimulus can be recovered from $\mathbf{y}$ and $\mathbf{w}_t$ by performing a pixel-wise shift of the image $\mathbf{y}$ by the amounts specified in $\mathbf{w}_t$.

  The eye movements are generated independently of the stimulus image during construction of the stimulus, and the reconstruction procedure correspondingly assumes that the image $\mathbf{y}$ and the eye movements $\mathbf{w}_{0:T}$ are independent.
- The encoding likelihood model captures retinal responses given knowledge of the full stimulus (e.g. with exactly known eye movements), and is parameterized as $p(\mathbf{s} \mid \mathbf{y}, \mathbf{w}_{0:T})$. For the LNBRC model presented in this paper, the encoding negative log-likelihood $-\log p(\mathbf{s} \mid \mathbf{y}, \mathbf{w}_{0:T})$ is convex in terms of the static image $\mathbf{y}$.

2 Reconstruction with known eye movements

To develop this approach, we first consider the simple case in which the eye movements $\mathbf{w}_{0:T}$ are known *a priori*. MAP reconstruction with known eye movements is formulated as

$$\begin{aligned}
\arg\max_{\mathbf{y}} \big\{ \log p(\mathbf{y} \mid \mathbf{s}) \big\} &= \arg\max_{\mathbf{y}} \big\{ \log p(\mathbf{y}, \mathbf{s}, \mathbf{w}_{0:T}) \big\} \\
&= \arg\max_{\mathbf{y}} \big\{ \log p(\mathbf{s} \mid \mathbf{y}, \mathbf{w}_{0:T}) + \log p(\mathbf{y}) + \log p(\mathbf{w}_{0:T}) \big\} \\
&= \arg\max_{\mathbf{y}} \big\{ \log p(\mathbf{s} \mid \mathbf{y}, \mathbf{w}_{0:T}) + \log p(\mathbf{y}) \big\}
\end{aligned}$$

where $\log p(\mathbf{w}_{0:T})$ is constant because $\mathbf{w}_{0:T}$ is known and can thus be discarded. Adding a weight λ on the prior term and flipping the sign yields the minimization problem

$$\arg\min_{\mathbf{y}} \big\{ -\log p(\mathbf{s} \mid \mathbf{y}, \mathbf{w}_{0:T}) - \lambda \log p(\mathbf{y}) \big\} \tag{1}$$

We solve the optimization problem in equation (1) approximately using a Plug-and-Play algorithm [1], leveraging the natural image prior implicit in a denoiser pre-trained to remove additive Gaussian noise from natural images. We specifically chose a half-quadratic splitting algorithm [2], which uses proximal variable splitting to split the full MAP optimization into likelihood and prior sub-problems, and alternates between the two. The likelihood sub-problem has form

$$\mathbf{x}^{(k)} = \arg\min_{\mathbf{x}} \{-\log p(\mathbf{s} \mid \mathbf{x}, \mathbf{w}_{0:T}) + \frac{\rho^{(k)}}{2} |\mathbf{x} - \mathbf{z}^{(k-1)}|_2^2\} \tag{2}$$

while the prior sub-problem has form

$$\mathbf{z}^{(k)} = \arg\min_{\mathbf{z}} \{-\lambda \log p(\mathbf{z}) + \frac{\rho^{(k)}}{2} |\mathbf{z} - \mathbf{x}^{(k)}|_2^2\}. \tag{3}$$

The likelihood sub-problem in equation (2) is an unconstrained convex minimization problem, and is solved using gradient descent. Because the prior sub-problem in equation (3) has the same objective as MAP Gaussian denoising with known additive noise variance $\lambda/\rho^{(k)}$, we compute its solution using a pre-trained convolutional neural network [2], resulting in the following approximate MAP algorithm:

Algorithm 1 Plug-and-Play approximate MAP for *known* eye movements

procedure PnP-Known-Eye-Movement-MAP($\mathbf{s}$, $\mathbf{w}_{0:T}$, $\mathbf{z}^{(0)}$)
  for $k \in 1, 2, ..., K$ **do**
    $\mathbf{x}^{(k)} \leftarrow \arg\min_{\mathbf{x}} \{-\log p(\mathbf{s} \mid \mathbf{x}, \mathbf{w}_{0:T}) + \frac{\rho^{(k)}}{2} |x - \mathbf{z}^{(k-1)}|_2^2\}$
    $\mathbf{z}^{(k)} \leftarrow$ Unblind-Denoiser-CNN($\mathbf{x}^{(k)}, \sigma^2 = \frac{\lambda}{\rho^{(k)}}$)
  end for
  return $\mathbf{z}^{(K)}$
end procedure

A variant of this algorithm for flashed stimuli (without eye movements) was previously presented in [3].

3 Reconstruction with unknown eye movements

In the case that the eye movements $\mathbf{w}_{0:T}$ are *unknown*, the MAP reconstruction problem requires marginalization over $\mathbf{w}_{0:T}$:

$$\begin{aligned}
\arg\max_{\mathbf{y}} \{ \log p(\mathbf{y} \mid \mathbf{s}) \} &= \arg\max_{\mathbf{y}} \{ \log p(\mathbf{y}, \mathbf{s}) \} \\
&= \arg\max_{\mathbf{y}} \left\{ \log \sum_{\mathbf{w}_{0:T}} p(\mathbf{y}, \mathbf{s}, \mathbf{w}_{0:T}) \right\} \\
&= \arg\max_{\mathbf{y}} \left\{ \log \sum_{\mathbf{w}_{0:T}} p(\mathbf{s} \mid \mathbf{y}, \mathbf{w}_{0:T}) p(\mathbf{w}_{0:T}) p(\mathbf{y}) \right\} \\
&= \arg\max_{\mathbf{y}} \left\{ \log p(\mathbf{y}) + \log \sum_{\mathbf{w}_{0:T}} p(\mathbf{s} \mid \mathbf{y}, \mathbf{w}_{0:T}) p(\mathbf{w}_{0:T}) \right\}.
\end{aligned}$$

The marginalization over possible eye movement trajectories is computationally intractable, and thus the exact MAP problem with eye movements cannot be directly solved. Expectation maximization (EM) is an

iterative algorithm can be applied to approximate the solution to the MAP problem while avoiding marginalization. The EM algorithm iteratively alternates between: (1) the *E-step*, constructing a lower bound on the negative log-posterior using a variational distribution on the eye movements $q^{(t)}(\mathbf{w}'_{0:T} \mid \mathbf{s}, \hat{\mathbf{y}}^{(t)})$; (2) the *M-step*, finding the intermediate image $\hat{\mathbf{y}}^{(t+1)}$ that maximizes the lower bound on the log-posterior constructed in the E-step; and (3) using $\hat{\mathbf{y}}^{(t+1)}$ to construct an updated variational distribution $q^{(t+1)}(\mathbf{w}'_{0:T} \mid \mathbf{s}, \hat{\mathbf{y}}^{(t)})$. Pseudocode outlining the iterative structure of the MAP-EM algorithm is provided in Algorithm 2. Descriptions of each of the steps of MAP-EM, as well as precise mathematical definitions for the subroutines PnP-Minimize-Expectation and Update-Q used in Algorithm 2 are provided in subsequent sections.

Algorithm 2 MAP-EM, iterating over timesteps (frame transitions)

```
procedure PnP-MAP-EM(s, q^(0)(w_{0:T}), y^(0))
    for t ∈ 0, 1, ..., T − 1 do
        y^(t+1) ← PnP-Minimize-Expectation(s, q^(t)(w_{0:T}), y^(0))        ▷ Image estimation
        q^(t+1)(w_{0:T}) ← Update-Q(s, y^(t+1), q^(t)(w_{0:T}))             ▷ Eye movement estimation
    end for
    return y^(T)
end procedure
```

Although this algorithm builds the eye movements trajectory distribution sequentially in time, it is *not* an online algorithm, because it assumes that all of the spikes in response to the stimulus presentation are available at every step of the computation (e.g. at every iteration, the algorithm integrates over spikes elicited by the entire stimulus presentation).

3.1 E-step: bounding the log-posterior

In order to construct the lower bound on the log-posterior on iteration $(t+1)$ of EM, we introduce a variational distribution $q^{(i)}(\mathbf{w}_{0:T} \mid \hat{\mathbf{y}}^{(t)}, \mathbf{s})$ on the eye movements. The variational distribution is allowed to depend on $\hat{\mathbf{y}}^{(t)}$,the guess for the image computed from the previous iteration of EM, as well as the observed spikes $\mathbf{s}$, and can take any form. Using Jensen's inequality, we construct a lower bound for the term $\log \sum_{\mathbf{w}_{0:T}} p(\mathbf{s} \mid \mathbf{y}, \mathbf{w}_{0:T}) p(\mathbf{w}_{0:T})$:

$$\begin{aligned}
\log \sum_{\mathbf{w}_{0:T}} p(\mathbf{s} \mid \mathbf{y}, \mathbf{w}_{0:T}) p(\mathbf{w}_{0:T}) &= \log \sum_{\mathbf{w}_{0:T}} p(\mathbf{s} \mid \mathbf{y}, \mathbf{w}_{0:T}) p(\mathbf{w}_{0:T}) \frac{q^{(t)}(\mathbf{w}_{0:T} \mid \hat{\mathbf{y}}^{(t)}, \mathbf{s})}{q^{(t)}(\mathbf{w}_{0:T} \mid \hat{\mathbf{y}}^{(i)}, \mathbf{s})} \\
&= \log \mathbb{E}_{\mathbf{w}_{0:T} \sim q^{(t)}(\mathbf{w}_{0:T} \mid \hat{\mathbf{y}}^{(t)}, \mathbf{s})} \left[\frac{p(\mathbf{s} \mid \mathbf{y}, \mathbf{w}_{0:T}) p(\mathbf{w}_{0:T})}{q^{(t)}(\mathbf{w}_{0:T} \mid \hat{\mathbf{y}}^{(t)}, \mathbf{s})} \right] \\
&\geq \mathbb{E}_{\mathbf{w}_{0:T} \sim q^{(t)}(\mathbf{w}_{0:T} \mid \hat{\mathbf{y}}^{(t)}, \mathbf{s})} \left[\log \frac{p(\mathbf{s} \mid \mathbf{y}, \mathbf{w}_{0:T}) p(\mathbf{w}_{0:T})}{q^{(t)}(\mathbf{w}_{0:T} \mid \hat{\mathbf{y}}^{(t)}, \mathbf{s})} \right] \\
&\geq \mathbb{E}_{\mathbf{w}_{0:T} \sim q^{(t)}(\mathbf{w}_{0:T} \mid \hat{\mathbf{y}}^{(t)}, \mathbf{s})} [\log p(\mathbf{s} \mid \mathbf{y}, \mathbf{w}_{0:T}) + \log p(\mathbf{w}_{0:T}) - \log q^{(t)}(\mathbf{w}_{0:T} \mid \hat{\mathbf{y}}^{(t)}, \mathbf{s})]
\end{aligned}$$

Thus the lower bound on the log-posterior constructed during the E-step is

$$\log p(\mathbf{y} \mid \mathbf{s}) \geq \log p(\mathbf{y}) + \mathbb{E}_{\mathbf{w}_{0:T} \sim q^{(i)}(\mathbf{w}_{0:T} \mid \mathbf{y}^{(i)}, \mathbf{s})} [\log p(\mathbf{s} \mid \mathbf{y}, \mathbf{w}) + \log p(\mathbf{w}_{0:T}) - \log q^{(i)}(\mathbf{w}_{0:T} \mid \mathbf{y}^{(i)}, \mathbf{s})] \tag{4}$$

3.2 M-step: maximizing the lower bound

The M-step maximizes the lower bound found in the E-step by finding the image $\hat{\mathbf{y}}^{(t+1)}$ that solves the optimization problem

$$\hat{\mathbf{y}}^{(t+1)} \triangleq \arg\max_{\mathbf{y}} \big\{ \log p(\mathbf{y}) + \mathbb{E}_{\mathbf{w}_{0:T}\sim q^{(t)}(\mathbf{w}_{0:T}|\hat{\mathbf{y}}^{(t)},\mathbf{s})}[\log p(\mathbf{s} \mid \mathbf{y}, \mathbf{w}_{0:T}) + \log p(\mathbf{w}_{0:T}) - \log q^{(i)}(\mathbf{w}_{0:T} \mid \hat{\mathbf{y}}^{(t)}, \mathbf{s})]\big\}.$$

The last two terms in the expectation are constants and can be discarded. After flipping the sign and turning the maximization problem into a minimization, we are left with

$$\mathbf{y}^{(t+1)} = \arg\min_{\mathbf{y}} \big\{ -\log p(\mathbf{y}) + \mathbb{E}_{\mathbf{w}_{0:T}\sim q^{(t)}(\mathbf{w}_{0:T}|\hat{\mathbf{y}}^{(t)},\mathbf{s})}[-\log p(\mathbf{s} \mid \mathbf{y}, \mathbf{w}_{0:T})]\big\}. \tag{5}$$

Because the encoding negative log-likelihood is convex in the image $\mathbf{y}$ for known spikes $\mathbf{s}$ and known eye movements $\mathbf{w}_{0:T}$, the expectation over $q^{(i)}$ is convex in $\mathbf{y}$. Thus, the optimization problem can be solved using the Plug-and-Play algorithm previously described, as long as the expectation is feasible to compute. Like before, we split the objective into likelihood and prior sub-problems using proximal variable splitting

$$\mathbf{x}^{(k)} = \arg\min_{\mathbf{x}} \{\mathbb{E}_{\mathbf{w}_{0:T}\sim q^{(t)}(\mathbf{w}_{0:T}|\hat{\mathbf{y}}^{(t)},\mathbf{s})}[-\log p(\mathbf{s} \mid \mathbf{x}, \mathbf{w}_{0:T})] + \frac{\rho^{(k)}}{2}|\mathbf{x} - \mathbf{z}^{(k-1)}|_2^2\} \tag{6}$$

$$\mathbf{z}^{(k)} = \arg\min_{\mathbf{z}} \big\{ -\lambda \log p(\mathbf{z}) + \frac{\rho^{(k)}}{2}|\mathbf{z} - \mathbf{x}^{(k)}|_2^2\big\} \tag{7}$$

and iteratively alternate between the subproblems, yielding the subroutine for the M-step

Algorithm 3 Plug-and-Play subroutine for EM (M-step)

procedure PnP-Minimize-Expectation($\mathbf{s}$, $q(\mathbf{w}_{0:T})$, $\mathbf{z}^{(0)}$)
  for $k \in 1, 2, ..., K$ **do**
    $\mathbf{x}^{(k)} \leftarrow \arg\min_{\mathbf{x}}\{\mathbb{E}_{\mathbf{w}_{0:T}\sim q(\mathbf{w}_{0:T})}[-\log p(\mathbf{s} \mid \mathbf{y}, \mathbf{w}_{0:T})] + \frac{\rho^{(k)}}{2}|\mathbf{x} - \mathbf{z}^{k-1)}|_2^2\}$
    $\mathbf{z}^{(k)} \leftarrow$ Unblind-Denoiser-CNN($\mathbf{x}^{(k)}, \sigma^2 = \frac{\lambda}{\rho^{(k)}}$))
  end for
  return $\mathbf{z}^{(K)}$
end procedure

3.3 Updating the variational distribution q

Finally, the variational distribution q is revised for the next iteration of MAP-EM using the observed spikes, $\mathbf{s}$, and the result of the M-step, $\hat{\mathbf{y}}^{(t+1)}$. In general, because construction of the lower bound on the log-posterior in the E-step does not depend on the form of q, q can take any form but must be a valid probability distribution. However, q should be near to the true conditional probability distribution $p(\mathbf{w}'_{0:T} \mid \mathbf{s}, \mathbf{y})$ in order for the lower bound to be tight.

The exact form of $p(\mathbf{w}'_{0:T} \mid \mathbf{s}, \mathbf{y})$ is first constructed. Let $\mathbf{w}_t$ denote the eye position at time t. Because a discretized 2D Brownian motion was used as the random process for generating eye movements for the stimulus, the eye position trajectory $\mathbf{w}_{0:T}$ language here satisfies the Markov property. Hence $p(\mathbf{w}'_{0:T}) = p(\mathbf{w}'_0) \prod_{i=1}^{T-1} p(\mathbf{w}'_i \mid \mathbf{w}'_{i-1})$. Using Bayes' rule, the true conditional distribution is

$$p(\mathbf{w}'_{0:T} \mid \mathbf{s}, \mathbf{y}) = \frac{p(\mathbf{s} \mid \mathbf{w}'_{0:T}, \mathbf{y}) p(\mathbf{w}'_0) \prod_{i=1}^{T-1} p(\mathbf{w}'_i \mid \mathbf{w}'_{i-1})}{p(\mathbf{s} \mid \mathbf{y})}. \tag{8}$$

The functional form of q was chosen to resemble equation (8):

$$q^{(t+1)}(\mathbf{w}'_{0:T}, \mid \mathbf{s}, \hat{\mathbf{y}}^{(t+1)}) \triangleq \frac{p(\mathbf{s} \mid \mathbf{w}'_{0:T}, \hat{\mathbf{y}}^{(t+1)}) r_0^{(t+1)}(\mathbf{w}'_0) \prod_{i=1}^{T-1} r_i^{(t+1)}(\mathbf{w}'_i \mid \mathbf{w}_{i-1})}{p(\mathbf{s} \mid \hat{\mathbf{y}}^{(t+1)})}. \tag{9}$$

The variational eye movement transition probabilities r_i can be arbitrarily chosen for computational convenience, and were chosen to be

$$r_0^{(t)}(\mathbf{w}_0) \triangleq \begin{cases} 1 & \text{if } \mathbf{w}_0 = \mathbf{0} \\ 0 & \text{otherwise} \end{cases} \tag{10}$$

$$r_i^{(t)}(\mathbf{w}_i \mid \mathbf{w}_{i-1}) \triangleq \begin{cases} p(\mathbf{w}_i \mid \mathbf{w}_{i-1}) & \text{if } i \le t \\ \begin{cases} 1 & \text{if } \mathbf{w}_i = \mathbf{w}_{i-1} \\ 0 & \text{otherwise} \end{cases} & \text{if } i > t \end{cases} \tag{11}$$

to enable massive cancellation (see next section). In words, the transition probabilities defined by(10) and (11) require that, on iteration t of the MAP-EM algorithm, the candidate eye position trajectory is required to start at the origin, is allowed to vary according to the two-dimensional Brownian motion for timesteps $1, 2, \ldots, t$, and is then held fixed at $\mathbf{w}_t$ for all subsequent timesteps $i > t$. Because the definitions of $r_i^{(t)}$ change depending on the iteration number t of MAP-EM, the definition of q changes on each iteration of MAP-EM.

3.3.1 Particle filter and sequential importance resampling

The primary criterion for choosing a representation of $q^{(t)}$ is that of computational complexity: the representation must be simple enough that the expectation in equation (6) can be evaluated efficiently. For this reason, particle filters are a particularly attractive option for representing $q^{(t)}$. Specifically, representing $q^{(t)}$ as a weighted collection of particles allows the expectation in equation (6) to be computed efficiently in parallel as a weighted sum over the particles. In addition, the total number of particles in the filter can be adjusted to trade-off accuracy in the representation of $q^{(t)}$ with the availability of computational resources.

The particle filter represents q as a set of candidate full eye movement trajectories and their associated probabilities. Specifically, on iteration t of MAP-EM, each of the particles $\gamma^{(p,t)} \triangleq (\mathbf{w}_{0:T}^{(p,t)}, \beta_p^{(t)})$ consists of a full candidate eye movement trajectory $\mathbf{w}_{0:T}^{(p,t)}$, and an associated probability $\beta_p^{(t)}$.

Sequential importance resampling [4] updates the particle filter for timestep $(t + 1)$ with the following steps: (1) sampling a new candidate trajectory for each particle $\mathbf{w}_{0:T}^{(p,t+1)}$ from a convenience distribution $v_{t+1}(\mathbf{w}_{0:T}^{(p,t+1)} \mid \mathbf{w}_{0:T}^{(p,t)})$; (2) re-weighting the probability for each particle with a multiplicative weight update $\alpha^{(p,t+1)}$ such that the overall particle filter faithfully represents the target sampling distribution; (3) resampling the particles such that each particle has equal weight, to ensure that the particle filter represents interesting parts of the distribution $q^{(t+1)}$, and to avoid numerical difficulties with small probabilities.

The particle-update sampling distribution is defined as

$$v_{t+1}(\mathbf{w}_{0:T}^{(t+1)} \mid \mathbf{w}_{0:T}^{(t)}) \triangleq \begin{cases} p(\mathbf{w}_{t+1} \mid \mathbf{w}_t = \mathbf{w}_t^{(t)}) & \text{if } \mathbf{w}_{t+2:T}^{(t+1)} = \mathbf{w}_{t+1} \text{ and } \mathbf{w}_{0,\ldots,t} = \mathbf{w}_{0,\ldots,t}^{(t)} \\ 0 & \text{otherwise} \end{cases}. \tag{12}$$

where $p(\mathbf{w}_{t+1} \mid \mathbf{w}_t)$ is the discretized Gaussian transition kernel used to generate the 2D Brownian motion. In words, this definition means that the trajectory for each particle is updated by setting the eye position at times $t + 1, t + 2, \ldots, T - 1$ to the value of a single random draw from $p(\mathbf{w}_{t+1} \mid \mathbf{w}_t)$, while leaving the eye positions from prior times $0, 1, \ldots, t$ unchanged.

The multiplicative weight update $\alpha^{(p,t+1)}$ is expressed as

$$\alpha^{(p,t+1)} = \frac{q^{(t+1)}(\mathbf{w}_{0:T}^{(t+1)} \mid \mathbf{s}, \hat{\mathbf{y}}^{(t+1)})}{q^{(t)}(\mathbf{w}_{0:T} \mid \mathbf{s}, \hat{\mathbf{y}}^{(t)}) v_{t+1}(\mathbf{w}_{0:T}^{(t+1)} \mid \mathbf{w}_{0:T}^{(t)})} \tag{13}$$

$$= \frac{p(\mathbf{s} \mid \mathbf{w}_{0:T}^{(t+1)}, \hat{\mathbf{y}}^{(t+1)}) r_0^{(t+1)}(\mathbf{w}_0^{(t+1)}) \prod_{i=1}^{T-1} r_i^{(t+1)}(\mathbf{w}_i^{(t+1)} \mid \mathbf{w}_{i-1^{(t+1)}})}{p(\mathbf{s} \mid \mathbf{w}_{0:T}^{(t)}, \hat{\mathbf{y}}^{(t)}) r_0^{(t)}(\mathbf{w}_0^{(t)}) \prod_{i=1}^{T-1} r_i^{(t)}(\mathbf{w}_i^{(t)} \mid \mathbf{w}_{i-1}^{(t)}) v_{t+1}(\mathbf{w}_{0:T}^{(t+1)} \mid \mathbf{w}_{0:T}^{(t)})} \tag{14}$$

Substituting in the definitions (10), (11), and (12) results in massive cancellation:

$$\begin{aligned}
\alpha^{(p,t+1)} &= \frac{p(\mathbf{s} \mid \mathbf{w}_{0:T}^{(t+1)}, \hat{\mathbf{y}}^{(t+1)})}{p(\mathbf{s} \mid \mathbf{w}_{0:T}^{(t)}, \hat{\mathbf{y}}^{(t)})} \cdot \frac{r_0^{(t+1)}(\mathbf{w}_0^{(t+1)}) \prod_{i=1}^{T} r_i^{(t+1)}(\mathbf{w}_i^{(t+1)} \mid \mathbf{w}_{i-1}^{(t+1)})}{v_{t+1}(\mathbf{w}^{(t+1)} \mid \mathbf{w}^{(t)}) r_0^{(t)}(\mathbf{w}_0^{(t)}) \prod_{i=1}^{T} r_i^{(t)}(\mathbf{w}_i^{(t)} \mid \mathbf{w}_{i-1}^{(t)})} \\
&= \frac{p(\mathbf{s} \mid \mathbf{w}_{0:T}^{(t+1)}, \hat{\mathbf{y}}^{(t+1)})}{p(\mathbf{s} \mid \mathbf{w}_{0:T}^{(t)}, \hat{\mathbf{y}}^{(t)})} \cdot \frac{p(\mathbf{w}_{t+1}^{(t+1)} \mid \mathbf{w}_t^{(t)}) \prod_{i=1}^{t} p(\mathbf{w}_i^{(t)} \mid \mathbf{w}_{i-1}^{(t)}) \prod_{i=t+2}^{T} r_i^{(t+1)}(\mathbf{w}_i^{(t+1)} \mid \mathbf{w}_{i-1}^{(t+1)})}{v_{t+1}(\mathbf{w}^{(t+1)} \mid \mathbf{w}^{(t)}) \prod_{i=1}^{t} p(\mathbf{w}_i^{(t)} \mid \mathbf{w}_{i-1}^{(t)}) \prod_{i=t+1}^{T} r_i^{(t)}(\mathbf{w}_i^{(t)} \mid \mathbf{w}_{i-1}^{(t)})} \\
&= \frac{p(\mathbf{s} \mid \mathbf{w}_{0:T}^{(t+1)}, \hat{\mathbf{y}}^{(t+1)})}{p(\mathbf{s} \mid \mathbf{w}_{0:T}^{(t)}, \hat{\mathbf{y}}^{(t)})} \cdot \frac{p(\mathbf{w}_{t+1}^{(t+1)} \mid \mathbf{w}_t^{(t)})}{v_{t+1}(\mathbf{w}^{(t+1)} \mid \mathbf{w}^{(t)})} \\
&= \frac{p(\mathbf{s} \mid \mathbf{w}_{0:T}^{(t+1)}, \hat{\mathbf{y}}^{(y+1)})}{p(\mathbf{s} \mid \mathbf{w}_{0:T}^{(t)}, \hat{\mathbf{y}}^{(t)})}
\end{aligned}$$

since both $\prod_{i=t+1}^{T} r_i^{(t)}(\mathbf{w}_i^{(t)} \mid \mathbf{w}_{i-1}^{(t)})$ and $\prod_{i=t+2}^{T} r_i^{(t+1)}(\mathbf{w}_i^{(t+1)} \mid \mathbf{w}_{i-1}^{(t+1)})$ each have value 1 where each respective distribution is supported. Hence α_{t+1} can be calculated by computing the ratio of encoding likelihood values, each using all of the spikes.

Pseudocode for the variational distribution update procedure is provided in Algorithm 4.

Algorithm 4 Variational distribution update subroutine

procedure UPDATE-Q($\mathbf{s}$, $\hat{\mathbf{y}}^{(t+1)}$, $\hat{\mathbf{y}}^{(t)}$, $q^{(t)}(\mathbf{w}_{0:T})$)
  $q^{(t+1)}(\mathbf{w}_{0:T}) \leftarrow [\,]$
  for particle $(\mathbf{w}_{0:T}^{(p,t)}, \beta^{(p,t)}) \in q^{(t)}(\mathbf{w}_{0:T})$ **do**
    $\mathbf{w}_{0:T}^{(t+1)} \leftarrow$ sample from $v_{t+1}(\mathbf{w}_{0:T}^{(t+1)} \mid \mathbf{w}_{0:T}^{(t)})$
    $\alpha_{p,t+1} \leftarrow \frac{p(\mathbf{s} \mid \hat{\mathbf{y}}^{(t+1)}, \mathbf{w}_{0:T}^{(t+1)})}{p(\mathbf{s} \mid \hat{\mathbf{y}}^{(t)}, \mathbf{w}_{0:T}^{(t)})}$
    Append $(\mathbf{w}_{0:T}^{(t+1)},\ \alpha_{p,t+1} \cdot \beta^{(p,t)})$ to $q^{(t+1)}(\mathbf{w}_{0:T})$
  end for
  $q^{(t+1)}(\mathbf{w}_{0:T}) \leftarrow$ RESAMPLE($q^{(t+1)}(\mathbf{w}_{0:T})$)
  return $q^{(t+1)}(\mathbf{w}_{0:T})$
end procedure

References

[1] S. V. Venkatakrishnan, C. A. Bouman, and B. Wohlberg, "Plug-and-Play priors for model based reconstruction," in *2013 IEEE Global Conference on Signal and Information Processing*, (Austin, TX, USA), pp. 945–948, IEEE, Dec. 2013.

[2] K. Zhang, Y. Li, W. Zuo, L. Zhang, L. Van Gool, and R. Timofte, "Plug-and-Play Image Restoration with Deep Denoiser Prior," *IEEE Transactions on Pattern Analysis and Machine Intelligence*, pp. 1–1, 2021.

[3] E. G. Wu, N. Brackbill, A. Sher, A. M. Litke, E. P. Simoncelli, and E. Chichilnisky, "Maximum *a posteriori* natural scene reconstruction from retinal ganglion cells with deep denoiser priors," in *Advances in Neural Information Processing Systems 35*, (New Orleans), May 2022.

[4] J. S. Liu and R. Chen, "Sequential Monte Carlo Methods for Dynamic Systems," *Journal of the American Statistical Association*, vol. 93, pp. 1032–1044, Sept. 1998.
